# *PIK3CA*-associated developmental disorders exhibit distinct classes of mutations with variable expression and tissue distribution

**DOI:** 10.1172/jci.insight.87623

**Published:** 2016-06-16

**Authors:** Ghayda Mirzaa, Andrew E. Timms, Valerio Conti, Evan August Boyle, Katta M. Girisha, Beth Martin, Martin Kircher, Carissa Olds, Jane Juusola, Sarah Collins, Kaylee Park, Melissa Carter, Ian Glass, Inge Krägeloh-Mann, David Chitayat, Aditi Shah Parikh, Rachael Bradshaw, Erin Torti, Stephen Braddock, Leah Burke, Sondhya Ghedia, Mark Stephan, Fiona Stewart, Chitra Prasad, Melanie Napier, Sulagna Saitta, Rachel Straussberg, Michael Gabbett, Bridget C. O’Connor, Catherine E. Keegan, Lim Jiin Yin, Angeline Hwei Meeng Lai, Nicole Martin, Margaret McKinnon, Marie-Claude Addor, Luigi Boccuto, Charles E. Schwartz, Agustina Lanoel, Robert L. Conway, Koenraad Devriendt, Katrina Tatton-Brown, Mary Ella Pierpont, Michael Painter, Lisa Worgan, James Reggin, Raoul Hennekam, Karen Tsuchiya, Colin C. Pritchard, Mariana Aracena, Karen W. Gripp, Maria Cordisco, Hilde Van Esch, Livia Garavelli, Cynthia Curry, Anne Goriely, Hulya Kayserilli, Jay Shendure, John Graham, Renzo Guerrini, William B. Dobyns

**Affiliations:** 1 Division of Genetic Medicine, Department of Pediatrics, University of Washington, Seattle, Washington, USA.; 2 Center for Integrative Brain Research and; 3 Center for Developmental Biology and Regenerative Medicine, Seattle Children’s Research Institute, Seattle, Washington, USA.; 4 Pediatric Neurology, Neurogenetics and Neurobiology Unit and Laboratories, Neuroscience Department, A. Meyer Children’s Hospital, University of Florence, Florence, Italy.; 5 Department of Genetics, Stanford University School of Medicine, Stanford, California, USA.; 6 Department of Medical Genetics, Kasturba Medical College, Manipal University, Manipal, Karnataka, India.; 7 Department of Genome Sciences, University of Washington, Seattle, Washington, USA.; 8 Whole Exome Sequencing Program, GeneDx, Gaithersburg, Maryland, USA.; 9 Regional Genetics Program, The Children’s Hospital of Eastern Ontario, Ottawa, Ontario, Canada.; 10 Department of Pediatrics, and Pediatric Neurology and Developmental Medicine, University Children’s Hospital, Tübingen, Germany.; 11 Mount Sinai Hospital, The Prenatal Diagnosis and Medical Genetics Division, Department of Obstetrics and Gynecology, and; 12 Department of Pediatrics, Division of Clinical and Metabolic Genetics, University of Toronto, Toronto, Ontario, Canada.; 13 Center for Human Genetics, University Hospitals Case Medical Center, Cleveland, Ohio, USA.; 14 Department of Pediatrics, Division of Medical Genetics, Saint Louis University, St. Louis, Missouri, USA.; 15 Department of Pediatrics, University of Vermont College of Medicine, Burlington, Vermont, USA.; 16 Department of Clinical Genetics, Royal North Shore Hospital, St Leonards, New South Wales, Australia.; 17 Belfast Health and Social Care Trust, Belfast, United Kingdom.; 18 Genetics, Metabolism and Pediatrics, London, Ontario, Canada.; 19 Clinical Genetics, Center for Personalized Medicine, Children’s Hospital Los Angeles, Keck School of Medicine at University of Southern California, Los Angeles, California, USA.; 20 Neurology Unit, Schneider Children’s Medical Center of Israel, Petach Tikva, and Sackler School of Medicine, Tel Aviv University, Tel Aviv, Israel.; 21 School of Medicine, Griffith University, Brisbane, Queensland, Australia.; 22 Division of Genetics, Department of Pediatrics, and; 23 Department of Human Genetics, University of Michigan, Ann Arbor, Michigan, USA.; 24 Genetics Service, Department of Pediatric Medicine, KK Women’s and Children’s Hospital, Singapore.; 25 British Columbia Medical Genetics Provincial Program, University of British Columbia, Vancouver, British Columbia, Canada.; 26 Service de génétique médicale, Centre Hospitalier Universitaire Vaudois CHUV, Switzerland.; 27 Greenwood Genetic Center, Greenwood, South Carolina, USA.; 28 Department of Dermatology, Children Hospital Prof. Dr. J. P. Garrahan, Buenos Aires, Argentina.; 29 Children’s Hospital of Michigan, Wayne State University, Detroit, Michigan, USA.; 30 Center for Human Genetics, University Hospitals Leuven and KU Leuven, Leuven, Belgium.; 31 South West Thames Regional Genetics Service, St George’s University NHS Foundation Trust, London, and Section of Cancer Genetics, Institute of Cancer Research, Sutton, United Kingdom.; 32 Department of Pediatrics and Ophthalmology, University of Minnesota, Minneapolis, Minnesota, USA.; 33 Department of Child Neurology, University of Florida, Jacksonville, Florida, USA.; 34 Department of Genetics, Liverpool Hospital, Liverpool, New South Wales, Australia.; 35 Department of Neurology, University of Washington, Seattle, Washington, USA.; 36 Providence Child Neurology, Providence Sacred Heart Medical Center and Children’s Hospital, Spokane, Washington, USA.; 37 Department of Pediatrics and Translational Genetics, Department of Pediatrics, Academic Medical Center, University of Amsterdam Medical Center, Amsterdam, The Netherlands.; 38 Department of Laboratories, Seattle Children’s Hospital and; 39 Department of Laboratory Medicine, University of Washington, Seattle, Washington, USA.; 40 División de Pediatría, Pontificia Universidad Católica de Chile, Pediatra-Genetista, Unidad de Genética, Hospital Dr. Luis Calvo Mackenna, Santiago, Chile.; 41 Department of Pediatrics, Sidney Kimmel Medical School at T. Jefferson University, Chief of Division of Medical Genetics, A.I. duPont Hospital for Children, Wilmington, Delaware, USA.; 42 Departments of Dermatology and Pediatrics, University of Rochester School of Medicine and Dentistry, Rochester, New York, USA.; 43 Center for Human Genetics, University Hospitals Leuven, KU Leuven, Leuven, Belgium.; 44 Clinical Genetics Unit, IRCCS Santa Maria Nuova Hospital, Reggio Emilia, Italy.; 45 University of California, San Francisco, San Francisco/Genetic Medicine Central California, San Francisco, California, USA.; 46 Weatherall Institute of Molecular Medicine, University of Oxford, Oxford, United Kingdom.; 47 Koç University, School of Medicine, Medical Genetics Department, Koç University Hospital, Istanbul, Turkey.; 48 Howard Hughes Medical Institute, Seattle, Washington, USA.; 49 Department of Pediatrics, Cedars-Sinai Medical Center, Harbor-UCLA Medical Center, David Geffen School of Medicine Los Angeles, California, USA.

## Abstract

Mosaicism is increasingly recognized as a cause of developmental disorders with the advent of next-generation sequencing (NGS). Mosaic mutations of *PIK3CA* have been associated with the widest spectrum of phenotypes associated with overgrowth and vascular malformations. We performed targeted NGS using 2 independent deep-coverage methods that utilize molecular inversion probes and amplicon sequencing in a cohort of 241 samples from 181 individuals with brain and/or body overgrowth. We identified *PIK3CA* mutations in 60 individuals. Several other individuals (*n* = 12) were identified separately to have mutations in *PIK3CA* by clinical targeted-panel testing (*n* = 6), whole-exome sequencing (*n* = 5), or Sanger sequencing (*n* = 1). Based on the clinical and molecular features, this cohort segregated into three distinct groups: (a) severe focal overgrowth due to low-level but highly activating (hotspot) mutations, (b) predominantly brain overgrowth and less severe somatic overgrowth due to less-activating mutations, and (c) intermediate phenotypes (capillary malformations with overgrowth) with intermediately activating mutations. Sixteen of 29 *PIK3CA* mutations were novel. We also identified constitutional *PIK3CA* mutations in 10 patients. Our molecular data, combined with review of the literature, show that *PIK3CA*-related overgrowth disorders comprise a discontinuous spectrum of disorders that correlate with the severity and distribution of mutations.

## Abstract 2

The clinical and molecular spectrum of PIK3CA-related developmental disorders are correlated with types of mutations, tissue distributions, and levels of mosaicism with the clinical phenotype.

## Introduction

Mosaicism refers to a biological phenomenon in which an individual derived from a single fertilized egg has two or more populations of cells with different genotypes. This process can be the result of spontaneous mutations occurring at different times during the life course of a multicellular organism, and is a widespread phenomenon during the normal aging process (1). Here, we will refer to somatic mosaicism as a process occurring strictly during development (postzygotically), excluding mosaicism confined to germ cells (germline mosaicism) and somatic mosaicism associated with cancer (2). While this phenomenon has been known for many years, the association of mosaic mutations with human disease began with discovery of mosaic chromosomal disorders, and patchy (or segmental) manifestations of Mendelian disorders (3, 4). The first sequencing-based proof of mosaic aberrations came in 1991 with discovery of activating mutations of the *GNAS* gene in McCune-Albright syndrome (5, 6). Subsequently, an increasing number of other conditions, largely congenital or childhood-onset developmental disorders, have been associated with mosaic mutations. Examples include Proteus syndrome (*AKT1*), Sturge-Weber syndrome (*GNAQ*), neurocutaneous melanosis (*NRAS*), epidermal nevi and linear nevus sebaceous syndrome (*HRAS*, *KRAS*, *PIK3CA*), Maffuci and Ollier syndromes (*IDH1*, *IDH2*), and encephalocraniocutaneous lipomatosis (*FGFR1*); disorders first proposed to be mosaic based on their asymmetric, patchy presentation, and lack of familial recurrence (7, 8). Mosaic mutations of these genes were primarily identified with the advent of massively parallel or next-generation sequencing (NGS) methods that facilitate detection of low-frequency variation, especially in affected tissues.

However, there are several limitations in our knowledge of mosaic developmental disorders, particularly which molecular diagnostic methods are best, which tissues should be assayed, and the nature and relationship between phenotypes and genotypes. The low-level mosaicism found in these disorders poses significant challenges to conventional clinical diagnostic approaches. Current molecular methods in standard clinical laboratories rely on Sanger sequencing for single-gene analysis, and standard-depth NGS methods including targeted panels or whole-exome sequencing (with many clinical diagnostic labs using 20× to 30× as the minimum depth of coverage for putatively constitutional or germline disorders) (9). However, these methods are not sensitive enough to detect low-level mutations, particularly when the level of mosaicism is very low (<5%–10%) in the tissue from which DNA is isolated (typically peripheral blood lymphocytes).

Of the genes associated with mosaicism in developmental disorders, mutations of *PIK3CA* have been associated with the widest spectrum of developmental phenotypes to date, with most described as distinct clinical entities long before the link to *PIK3CA* was discovered (10). *PIK3CA* encodes the alpha catalytic subunit of phosphatidylinositol-4,5-bisphosphate 3-kinase, a central member of the phosphatidylinositol 3-kinase (PI3K) enzyme family (11). *PIK3CA* functions as an oncogene, and activating (or gain-of-function) mutations of *PIK3CA* are widely seen in human cancers (12, 13). The most common *PIK3CA* mutations are p.Glu542Lys, p.Glu545Lys, and p.His1047Arg, which are seen in ~80% of somatic tissues in cancer (and therefore termed “hotspot” mutations). Based on their degree of activity, several other classes of *PIK3CA* mutations have been described including strong, intermediate, and weak (13). Some of these same gain-of-function *PIK3CA* mutations have been recently reported in a range of pediatric developmental phenotypes. These disorders are broadly characterized by cutaneous vascular malformations with segmental overgrowth and involve multiple tissues or body regions. These conditions have been variably classified as Klippel-Trenaunay (KTS), congenital lipomatosis with overgrowth, vascular malformations, epidermal nevi, and skeletal/scoliosis/spinal abnormalities (CLOVES), megalencephaly-capillary malformation syndrome (MCAP), and dysplastic megalencephaly (DMEG), but the spectrum and differences between these disorders have not been defined.

Here, we report the largest series of patients to date with developmental disorders associated with *PIK3CA* mutations, identified using 2 orthogonal deep-targeted NGS methods that utilize molecular inversion probes (MIPs) and amplicon sequencing, to determine the mutational spectrum and quantify mutation levels in multiple tissues from affected individuals. By combining our data with previous reports, we assess available clinical molecular diagnostic methods, the most ideal tissue samples to be assayed, and derive genotype-phenotype correlations in this spectrum of disorders. We show that the phenotypes are not simply related to the level and distribution of *PIK3CA* mutations, but also to the class of mutation. Specifically, we show that CLOVES and KTS, as well as most localized lesions, are caused by one of the three most common oncogenic (“hotspot”) mutations or occasionally by strong or intermediate mutations, while MCAP is caused largely by a different set of less-activating mutations, with a much wider mutational spectrum that overlaps at only a few strong mutations (13). We also show that mutations are not equally detectable from apparently unaffected but easily available “surrogate” tissues, with levels of mosaicism on average lower in peripheral blood lymphocytes than saliva, and lower in saliva than skin fibroblasts.

## Results

We screened a cohort of 241 DNA samples from 181 individuals with brain and body overgrowth for mutations in the *PIK3CA* gene using two deep NGS methods that utilize MIPs (14, 15) and amplicon sequencing. This cohort included 131 individuals with features of MCAP, 19 with diffuse brain overgrowth or megalencephaly, and 31 with various forms of somatic overgrowth and vascular malformations including CLOVES, KTS, and capillary malformations with overgrowth (Table 1 and Supplemental Figure 1; supplemental material available online with this article; doi:10.1172/jci.insight.87623DS1). We identified mutations of *PIK3CA* in 60 individuals (33%). Several other patients (*n* = 12) were identified to have mutations separately by clinical targeted-panel testing (*n* = 6), clinical whole-exome sequencing (*n* = 5), or Sanger sequencing (*n* = 1), and were included to highlight the clinical and molecular variability of this spectrum of disorders. This cohort includes one of our previously published MCAP patients, as we have analyzed several newly acquired tissue samples from this patient in this study (16). The remaining 23 patients with MCAP who were previously published were excluded from this study (16). The mutations identified, levels of mosaicism, and tissue distribution in this cohort are listed in Tables 2, 3, and 4. The levels of mosaicism in all tissues of all mutation-positive patients and their parents using all molecular methods are listed in Supplemental Table 1.

### Molecular results

#### Levels of mosaicism and tissue distribution.

**Figure 1:**
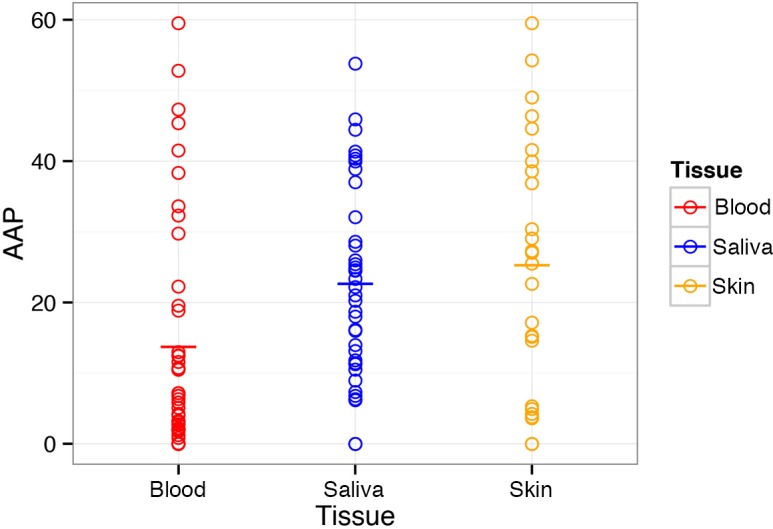
Levels of mosaicism in *PIK3CA* by sample type. Dot blot graph showing alternative allele percentages (AAPs) clustered by type of tissue in all mutation-positive individuals (*n* = 72). Horizontal bars indicate the mean AAP within each sample type: red = blood (*n* = 44); blue = saliva (*n* = 38); orange = skin fibroblasts (*n* = 26). Two-tailed *t* test (*P* values): blood-saliva: *P* = 0.035; blood-skin: *P* = 0.036; saliva-skin, *P* = 0.65.

We identified 29 mutations of *PIK3CA*, including 16 novel mutations that have not been previously identified in developmental disorders, to our knowledge. Oncogenic mutations at all of these amino acid residues were present in the Catalogue of Somatic Mutations in Cancer (COSMIC; http://cancer.sanger.ac.uk/cosmic). None of the mutations were present in public databases including the 1000 Genomes project (http://www.1000genomes.org), the NHLBI Exome Variant Server (http://evs.gs.washington.edu/EVS), or the Exome Aggregation Consortium (ExAC; http://exac.broadinstitute.org), with the exception of p.Glu545Lys and p.His1047Arg, which were present at very low frequencies in ExAC (<0.0001). Alternative allele percentages (the percentage of alternate or mutant reads to total reads; AAP) ranged from 1% to 59.5% across the entire cohort (Tables 2, 3, and 4 ). We observed substantial variation in AAPs in multiple samples from the same individual, with levels of mosaicism in blood (peripheral lymphocytes) ranging from undetectable to 59.5% (mean AAP 16.5%; 43 samples), saliva (lymphocytes and epithelial cells) from undetectable to 54% (mean AAP 24%; 37 samples), and skin-derived fibroblasts from 4% to 60% (mean AAP 26.4%; 20 samples). The AAPs grouped by sample type (blood, saliva, skin fibroblasts) are shown in Figure 1, whereas the AAPs for each sample type in all mutation-positive individuals are shown in Supplemental Figure 2. For a few individuals, we tested additional sample types including buccal swabs (*n* = 4 samples), surgically resected tonsils (lymphoid tissue; *n* = 2), and occipital bone dura mater removed during posterior fossa decompression surgery (*n* = 1).

#### Diagnostic testing methods.

The average coverage of single-molecule MIPs (smMIPs) was 450.74×, with an average of 71.27% of coding regions covered by 50 or more capture events, and with 2% being the lower threshold of mutant allele detection. All amino acid residues in which mutations were previously identified were well covered by smMIPs in the entire cohort (Supplemental Figure 3). All mutations identified via smMIPs were confirmed using a second orthogonal method (amplicon sequencing), when sufficient DNA was available. Amplicon sequencing yielded high coverage data for all mutations, with a mean coverage of 1975×. None of these mutations were present in the parents of affected individuals by amplicon sequencing. Overall, AAPs detected by amplicon sequencing were similar to smMIP results, although the latter were usually slightly higher for the same samples (Supplemental Figure 4). This may be partly due to allelic bias/preferential amplification of amplicon sequencing when compared to smMIPs.

Clinical testing identified several more patients with *PIK3CA* mutations. Mutations in 6 individuals were detected by targeted NGS using the Agilent SureSelect Capture technology. Another 5 were identified using standard-depth whole-exome sequencing (WES) on blood-derived DNA, and 1 patient was identified to have a mutation by standard Sanger sequencing. AAPs in these samples ranged from 41%–59.5%, suggesting constitutional mutations, with the exception of 1 patient (LR15-238) found to have the p.Arg93Gln mutation in 30 of 159 reads (19%). AAPs in the 6 patients tested by targeted NGS ranged from 1%–47%. In 2 patients (LR14-323, LR15-227), AAPs were 47% in blood-derived DNA, again suggesting constitutional mutations.

**Figure 2:**
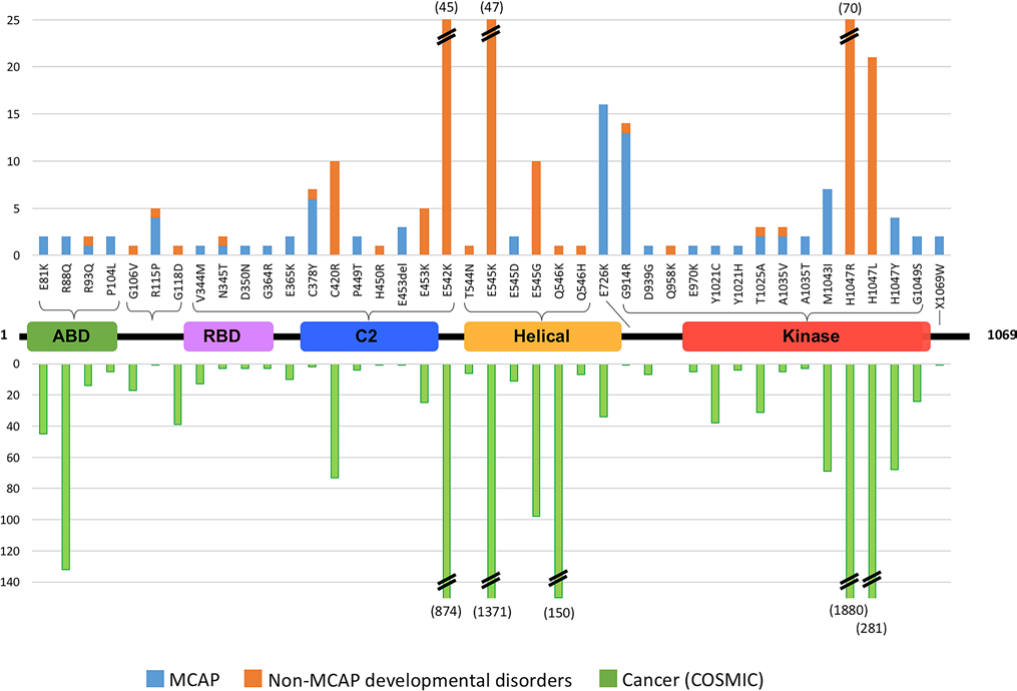
Distribution of *PIK3CA* mutations by functional domain in cancer and developmental (pediatric) disorders. Graph showing the number of published *PIK3CA* mutations by amino acid location in the Catalogue of Somatic Mutations in Cancer (COSMIC) database of somatic variation in cancer (shown in green; last accessed May 2016) and in children with developmental disorders comparing the megalencephaly-capillary malformation syndrome (MCAP; shown in blue) and all other developmental disorders (shown in orange). Mutations shown include those reported in this study as well as published mutations. Notes: (a) The 2-tailed *P* value by Fisher’s exact test was less than 0.0001, supporting that the association between MCAP and non-hotspot mutations and non-MCAP and hotspot associations is extremely statistically significant. (b) “Hotspot” mutations in this analysis are the most activating mutations in somatic tissues in cancer (p.Glu542Lys, p.Glu545Lys, p.His1047Arg) (13).

Overall, we identified 29 *PIK3CA* mutations in 72 individuals. We also reviewed published data on *PIK3CA* mutations in developmental pediatric disorders (all phenotypes except for cancer; Figure 2) (16–29). When added to our data, we find that all developmental phenotypes combined have been associated with 41 different *PIK3CA* mutations involving 33 codons and include 27 recurrent mutations (i.e., seen in more than one affected individual). The three hotspot *PIK3CA* mutations (p.Glu542Lys, p.Glu545Lys, and p.His1047Arg) are highly recurrent (i.e., seen frequently) in pediatric disorders. We found that two “non-hotspot mutations” (p.Glu726Lys and p.Gly914Arg) are also highly recurrent in developmental phenotypes, having now been detected in more than 10 patients each.

Most of these mutations are proven or predicted to have a gain-of-function (GOF) mechanism, and oncogenic mutations at all of these amino acid sites have been seen in COSMIC (13). Published functional studies demonstrate a GOF mechanism for at least 9 of 41 mutations. For another 8 mutations, GOF has been shown for different missense mutations at the same codon (13). For example, our study identified p.Asn345Thr, p.Glu545Asp, and p.Gln546His mutations, while GOF has been shown for p.Asn345Lys, p.Glu545Lys, p.Glu545Gly, p.Gln546Lys, and p.Gln546Pro. We also detected p.Tyr1021His and p.Ala1035Thr mutations in our series, while p.Tyr1021Cys and p.Ala1035Val mutations have been found in other patients (16). Further, Glu545Ala and Glu545Lys mutations have been previously detected in individuals with undefined “Cowden-like” features as well (30).

### Clinical results

**Figure 3:**
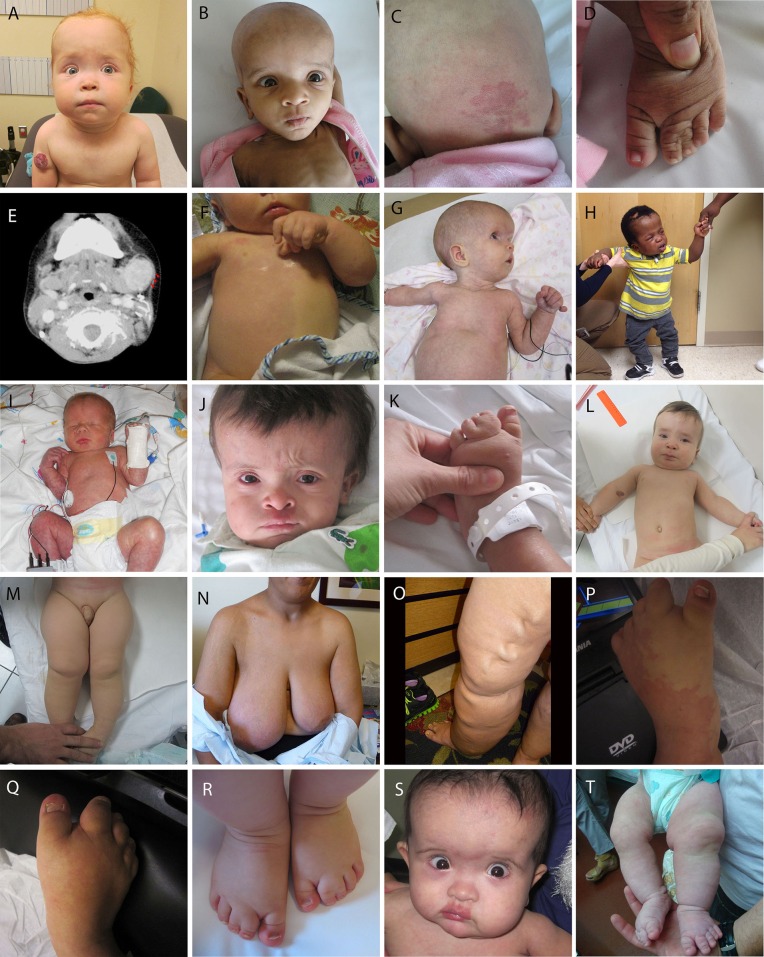
Clinical photographs of MCAP patients. (**A**) Facial photograph of patient LR14-323 (p.Arg83Gln). (**B**–**D**) Photograph of the face (**B**), occipital region (**C**), and left foot (**D**) of LR13-359 (p.Pro104Leu) showing MEG, occipital capillary malformation, and syndactyly of the second, third, and fourth toes. (**E**) Computed tomography (CT) image of LR01-060 (p.Pro104Leu) showing the subcutaneous hemangioma (arrowheads). (**F**) Photograph of the trunk of LR12-080 (p.Arg115Pro) showing cutaneous capillary malformation with midline delineation. (**G**) Photograph of LR12-365 (p.Asn345Thr) showing diffuse capillary malformations, MEG with a prominent forehead, and postaxial polydactyly of the left hand. (**H**) Photograph of LR13-036 (p.Glu365Lys) showing MEG, a disproportionately small body, and short extremities (clinically diagnosed with rhizomelic shortening of the extremities). (**I**) Photograph of LR11-418 (p.Cys378Tyr) showing diffuse capillary malformations and apparent megalencephaly (MEG). (**J** and **K**) Photograph of the face (**J**) and left foot (**K**) of LR12-330 (p.Glu545Asp) showing clear MEG, capillary malformation of the philtrum, skin laxity of the forehead, and syndactyly of the second, third, and fourth toes. (**L** and **M**) Photographs of the face (**L**) and body (**M**) of LR13-038 (p.Gly914Arg) showing MEG, reticulated capillary malformations, pigmented nevus of the right arm, and asymmetry of the legs. (**N** and **O**) Photographs of the chest (**N**) and lower extremity (**O**) of LR12-383 (p.Gly914Arg) showing clear asymmetry of the trunk involving the right breast and overgrowth of the right leg with prominent venous network. (**P** and **Q**) photographs of the left (**P**) and right (**Q**) feet of LR11-081 (p.Thr1025Ala) showing bilateral asymmetric macrodactyly, sandal-gap toes, and capillary malformations. (**R**) Photograph of the feet of LR13-169 (p.Ala1035Thr) showing syndactyly of the second, third, and fourth toes on the right, and second and third toes on the left. (**S** and **T**) photographs of the face (**S**) and lower extremities (**T**) of LR12-328 (p.Met1043Ile) showing facial and body asymmetry, MEG with a prominent forehead, and capillary malformations on the face and body.

**Figure 4:**
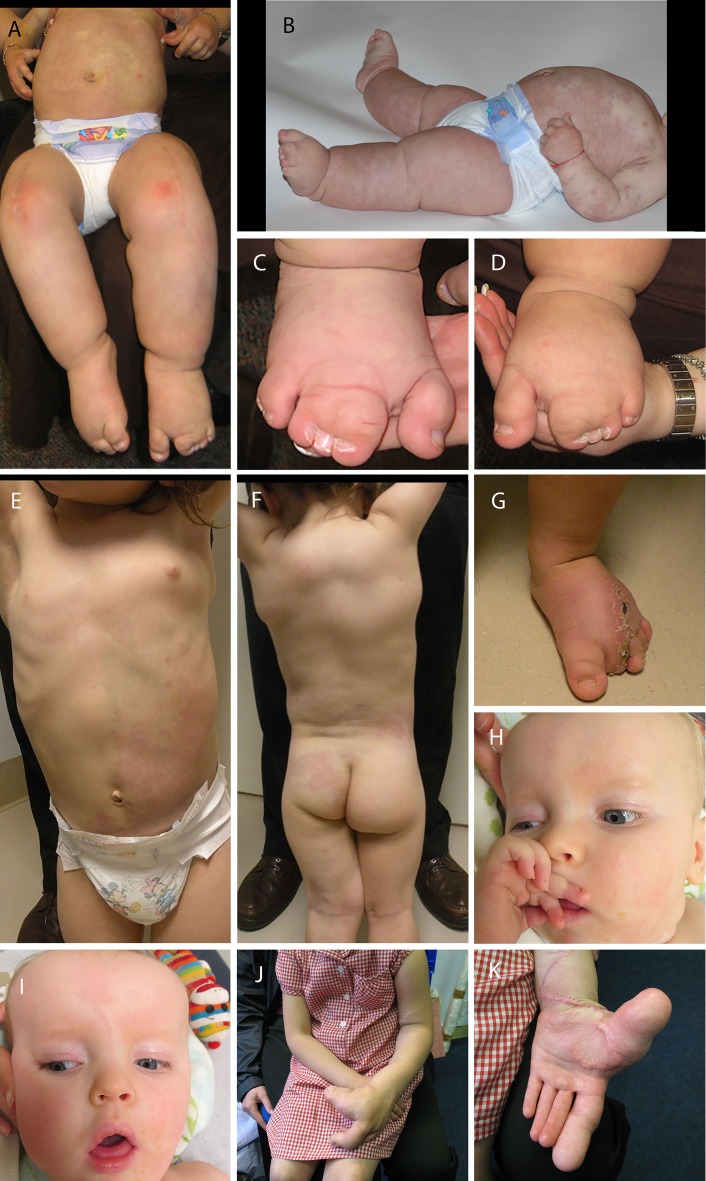
Clinical photographs of *PIK3CA* mutation–positive patients with segmental overgrowth. (**A**–**D**) Photographs at 1 year (**A**), birth (**B**), right foot (**C**), and left foot (**D**) of LR11-082 (p.Gly106Val) showing diffuse and asymmetric body overgrowth, diffuse capillary malformations, bilateral syndactyly of second, third, and fourth toes, and joint hypermobility. (**E**–**G**) Photograph of the body (**E** and **F**) and left foot (**G**) of LR12-070 (Glu453Lys) showing asymmetric overgrowth, capillary malformations with midline delineation, and postsurgical changes after resection of the second toe due to severe macrodactyly. (**H** and **I**) Facial photographs of patient LR12-183 (p.Glu542Lys) showing multiple epidermal nevi. (**J** and **K**) Photographs of LR12-172 (p.His1047Arg) showing macrodactyly of the left hand.

**Figure 5:**
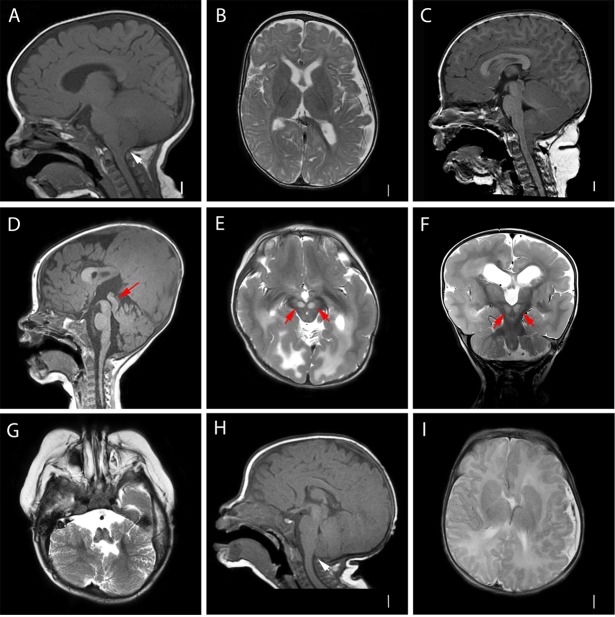
Brain MRI images of *PIK3CA* mutation–positive patients. (**A** and **B**) LR11-082. T1-weighted mid-sagittal (**A**) and T2-weighted axial (**B**) image at age 6 months showing a large cerebellum, crowded posterior fossa with cerebellar tonsillar ectopia, and a relatively normal cortical gyral pattern (arrowhead). (**C**) LR12-184. T1-weighted mid-sagittal image showing marked cerebellar tonsillar ectopia (arrowhead). (**D**–**H**) LR12-183. T1-weighted mid-sagittal (**D**), T2-weighted axial (**E** and **G**), and coronal (**F**) images showing diffuse cortical dysplasia, partial agenesis of the corpus callosum, dysplasia/hypoplasia of the cerebellar vermis and hemispheres, diffuse dysmyelination, abnormal high T2 signal intensities in the red nuclei and thalami bilaterally (red arrows in **E** and **F**), and a very large tectum (red arrow in **D**). (**H** and **I**) LR13-197. T1-weighted mid-sagittal (**H**) and T2-weighted axial (**I**) images showing bilateral cortical dysplasia, hippocampal dysplasia, white matter dysmyelination, and bilaterally dysplastic ventricles. There is mild cerebellar tonsillar ectopia (arrow in **H**).

The clinical features of all mutation-positive individuals are listed in Supplemental Tables 2 and 3. Clinical photographs and brain MRI images of some of these individuals are shown in Figures 3–5. Based on clinical and molecular characteristics, these patients can be segregated into three groups, as follows.

#### Group 1: MCAP syndrome.

Most individuals with mosaic *PIK3CA* mutations in our cohort (*n* = 50) had classic features of MCAP, characterized by brain overgrowth (megalencephaly) and cutaneous vascular malformations, with variable body overgrowth, connective tissue laxity, and digital anomalies (polydactyly and syndactyly) (Figure 3) (31, 32). Several of our MCAP patients had additional noteworthy clinical findings including (a) complex structural heart defects or arrhythmias (*n* = 12), (b) lymphatic malformations including chylothorax and lymphedema (*n* = 5), (c) predisposition to thrombosis (*n* = 3), (d) endocrine disorders including hypothyroidism (*n* = 3), growth hormone deficiency (*n* = 2), and rhizomelic shortening of the extremities (*n* = 3). Six patients had episodes of hypoglycemia (Supplemental Tables 2 and 3). Interestingly, several of our MCAP patients (*n* = 6) had constitutional (or apparently constitutional) mutations in *PIK3CA* (including LR14-323, LR14-278, LR14-358, LR13-048, LR15-227, and LR15-246). Two of these patients (LR15-227 and LR15-246) had the p.Glu726Lys mutation.

#### Group 2: intermediate phenotypes — capillary malformations with overgrowth and lipomatosis (CMO, CMOL).

Several patients (*n* = 7) had novel phenotypes with features either overlapping MCAP but lacking megalencephaly or suggesting a milder variant of CLOVES. Patient LR15-238 had mild somatic hemihypertrophy and cutaneous vascular malformations (facial capillary malformation, and reticulated diffuse capillary malformations on the body), but was normocephalic. She had perinatal bradycardia, transient dilated cardiomyopathy, and a history of possible nonocclusive venous thrombosis in the internal jugular vein of unknown etiology. She had the p.Arg93Gln mutation in 19% of alleles in peripheral blood lymphocytes. Interestingly, the same mutation was identified in another patient with classic features of MCAP (LR14-323). The mutation in the latter patient appeared to be constitutional, seen in ~50% of alleles in blood- and saliva-derived DNA. Patient LR11-082 had congenital onset somatic overgrowth, diffuse congenital livedo reticularis and syndactyly, as well as severe joint laxity and multiple joint dislocations (Figure 4, A–D). She was normocephalic, and brain MRIs performed at 5 days and 6 months of age showed no cortical malformation. Interestingly, she had progressive cerebellar tonsillar ectopia and a markedly large cerebellum on her MRI at 6 months (Figure 5, A and B). This patient harbored the p.Gly106Val mutation in 34% and 40% of alleles in blood and skin, respectively. Patient LR12-131 had hemihypertrophy, capillary malformation on the philtrum and midline lower lip, a capillary malformation on the right arm, and toe syndactyly, but lacked other classic features of MCAP. He had a mosaic p.Cys378Tyr mutation in 7% of alleles in blood-derived DNA.

Patient LR12-070 had congenital hemihypertrophy, epidermal nevi, and lipomatous overgrowth of the trunk that was characteristically milder than CLOVES (Figure 4, E–G). He also had overlying capillary malformations, and macrodactyly requiring surgical excision. He harbored the p.Glu453Lys mutation in 25% of alleles in fibroblasts. This mutation was undetectable in saliva. Patient LR12-184 had diffuse asymmetric somatic overgrowth, reticulated port-wine stain, syndactyly, and connective tissue laxity. He was normocephalic, with no cortical malformations, and normal cognition. Interestingly, his brain MRI scan at age 2 months showed severe cerebellar tonsillar ectopia (Figure 5C). He subsequently developed sporadic swelling of the neck believed to be due to lymphatic fluid accumulation. He harbored the p.Glu453Lys mutation at variable levels (ranging from 5%–17% of alleles in affected skin regions, 24.5% in saliva, and 0.9% of alleles in blood-derived DNA). Patient LR12-329 had hemihypertrophy, cutaneous vascular malformations, and multiple cutaneous lipomas, but lacked classic features of MCAP or CLOVES. He had the p.Glu453Lys mutation in 6% of alleles in saliva. Patient LR13-045 also had hemihypertrophy and diffuse congenital livedo reticularis but lacked megalencephaly. He had the p.Gly914Arg mutation in 5% and 15% of alleles in normal and affected skin-derived fibroblasts, respectively. This mutation was undetectable in saliva-derived DNA.

#### Group 3: hotspot-associated phenotypes (CLOVES/DMEG).

Four patients harbored hotspot *PIK3CA* mutations most commonly seen in cancer. Two of these patients had a mosaic p.Glu542Lys mutation. Patient LR12-183 had a novel constellation of features including megalencephaly with diffuse cortical dysplasia, partial agenesis of the corpus callosum, dysplasia/hypoplasia of the cerebellar vermis and hemispheres, diffuse dysmyelination, abnormal high T2 signal intensities in the red nuclei and thalami bilaterally, and a very large tectum (Figure 5, D–G). He also had multiple linear sebaceous nevi on the face and trunk (Figure 4, H and I). This combination of features meets criteria for linear nevus sebaceous or Schimmelpenning syndrome (33–35). This patient’s mutation was seen in 39/139 (28%) of alleles in saliva-derived DNA. Patient LR13-197 had diffuse megalencephaly with bilateral cortical dysplasia, hippocampal dysplasia, white matter dysmyelination, and dysplastic ventricles bilaterally (Figure 5, H and I). She harbored the p.Glu545Lys mutation at 26% of alleles in saliva-derived DNA. Patients LR13-172 and LR13-265 both had very low-level mosaic p.His1047Arg mutations. Patient LR13-172 had megalencephaly with macrodactyly and harbored the mutation in 1% of alleles in blood-derived DNA (Figure 4, J and K). Patient LR13-265 had features of CLOVES syndrome with lipomatous overgrowth involving the face and trunk, prominent venous network in the abdominal wall, verrucous overgrowth of the trunk, and macrodactyly. His mutation was present in 4% of affected skin-derived DNA, and absent from several unaffected skin samples. We also obtained additional information on a previously reported patient (LR12-033) (16, 36) who demonstrates features of both dysplastic megalencephaly (effectively bilateral hemimegalencephaly) and CLOVES syndrome, making her only the second reported child with this severe combination. She had severe asymmetric overgrowth, diffuse soft tissue swelling consistent with lymphedema, asymmetric head, severe hypotonia, and possible right optic nerve hypoplasia (Supplemental Figure 5).

#### Constitutional PIK3CA mutations.

Ten individuals in our cohort harbored constitutional (or apparently constitutional) mutations of *PIK3CA* that were detectable in peripheral samples (blood or saliva) at AAPs ranging from 35%–60% (LR14-323, LR01-060, LR15-076, LR13-036, LR14-278, LR14-358, LR13-048, LR15-227, LR12-330, LR15-246, and LR12-340). Interestingly, seven of these individuals had features of MCAP. However, three had diffuse brain or body overgrowth without segmental or patchy manifestations. Patient LR15-076 had diffuse megalencephaly, a small patent foramen ovale, and pectus excavatum deformity. Patient LR13-036 had a unique constellation of features including megalencephaly with rhizomelic shortening of the extremities, polydactyly, complex cardiac defects (left-sided aortic arch, ventricular septal defect, atrial dilatation), recurrent hypoglycemia, omphalocele, organomegaly, laryngeal stenosis, and recurrent infections, partially overlapping with Beckwith-Wiedemann syndrome. Patient LR12-340 had somatic asymmetry, was tall for age, with bilateral 2-3 toe syndactyly, but lacked megalencephaly and vascular anomalies. Mutations in five of these individuals were detected by clinical WES. These *PIK3CA* mutations were detected in blood at standard coverage (20× to 30×), suggesting constitutional mutations. Of note, blood-derived DNA was not available for analysis on four of these individuals (LR12-340, LR01-060, LR11-200, and LR12-330). Therefore, the possibility of mosaicism cannot be excluded in these patients.

## Discussion

### Classification of PIK3CA-associated developmental phenotypes.

**Figure 6:**
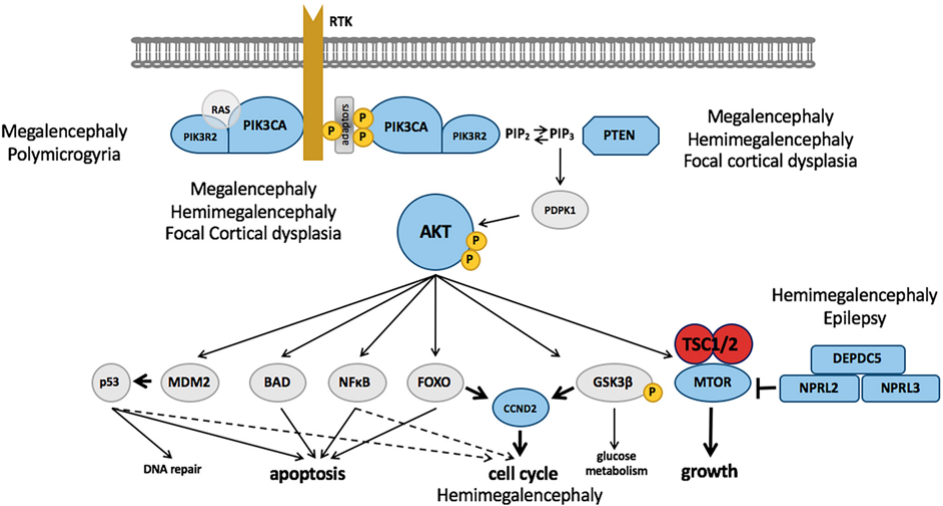
The PI3K-AKT-MTOR–related developmental brain disorders spectrum. Diagram showing the PI3K-AKT-MTOR pathway highlighting genes associated with developmental brain disorders including *PIK3CA*, *PIK3R2*, *PTEN*, *AKT3*, *MTOR*, *CCND2*, *DEPDC5*, *NPRL2*, and *NPRL3* (shown in blue), as well as *TSC1* and *TSC2* genes (shown in red).

GOF mutations of *PIK3CA* have been associated with a wide spectrum of pediatric phenotypes (Figure 6). The original and best known of these is KTS, which was first defined more than a century ago based on the now classic triad of capillary malformation, venous varicosities, and limb hypertrophy (37–39). However, published photographs of KTS vary considerably in appearance. Therefore, it appears that KTS is often used as an umbrella term for any disorder with vascular malformation and overgrowth. Several recently described syndromes appear to be more specific designations, and we have predominantly used these in analyzing our phenotypic data. CLOVES is the most severe KTS-like disorder. In practice, most patients with CLOVES have all of the anomalies in the mnemonic (above), dramatic postnatal progression of the segmental overgrowth and lipomatosis, and other abnormalities (19, 40, 41). A subset of patients with most but not all of the features of CLOVES has been designated as fibroadipose overgrowth/hemihyperplasia-multiple lipomatosis, but the criteria used to separate them are unclear (10, 21). Capillary malformation with overgrowth (CMO) is a recently defined clinical entity characterized by extensive, diffuse, reticulate capillary malformations and variable hypertrophy without major complications (42). The phenotype is much less severe than CLOVES, although some patients with apparent CMO in infancy go on to develop lipomatosis at older ages. Several other relatively mild phenotypes that overlap with CMO have also been described, including the “girth” and “RCM” types of vascular malformations with overgrowth (43). MCAP is characterized by symmetric or only mildly asymmetric brain overgrowth (megalencephaly) and cutaneous vascular malformations (predominantly capillary malformations), with variable malformations of cortical development (polymicrogyria), body overgrowth, digital anomalies (syndactyly and less often polydactyly), and connective tissue laxity (31, 32, 44, 45).

Beyond these multisystem disorders, mosaic mutations of *PIK3CA* have recently been identified in localized or focal forms of overgrowth with dysplasia including epidermal nevi (17), isolated macrodactyly (22), infiltrating lipomatosis (25, 46), isolated lymphatic malformations (28), and, most recently, venous malformations (29). Further, several patients with focal brain involvement, including dysplastic megalencephaly (DMEG), hemimegalencephaly (HMEG), and focal cortical dysplasia (FCD) have been identified as well (20, 27, 36). These disorders have been associated with variable but often low levels of mutations in affected tissues, and very low or undetectable levels in apparently unaffected tissues, such as peripheral blood lymphocytes. Finally, several patients have been reported with constitutional mutations of *PIK3CA* associated with isolated megalencephaly or unspecified “Cowden-like” features (30, 47).

Several recent reviews have presented *PIK3CA*-associated phenotypes as essentially a single broad spectrum (10). However, the original phenotype descriptions and the molecular data presented here argue otherwise. We view the differences between the clinical presentation as consistent and recognizable, based on our observations in the largest series of mosaic mutations of a single gene underlying developmental disorders reported to date. We propose that the *PIK3CA*-associated spectrum of disorders should serve as a paradigm for mosaic disorders. The clinical differences between MCAP and CLOVES (including CLOVES-DMEG) syndromes are summarized in Table 5. In MCAP, head and brain overgrowth are often the presenting signs, while body overgrowth at birth is mild (+1 to +2 SD) with stature normalizing by ~8 years (32). The cutaneous vascular malformation typically presents as congenital livedo reticularis (38). The head and body overgrowth and livedo reticularis are most often diffuse with subtle asymmetry. About 60%–70% of patients have a cortical malformation, with imaging and clinical features consistent with bilateral perisylvian polymicrogyria (31, 32). Lipomas, lymphatic malformations, and macrodactyly occur, but are typically uncommon, limited in size, do not progress, and rarely require surgical intervention. Epidermal nevi are rare. In CLOVES, on the other hand, head and brain size are typically normal, although rare patients with combined CLOVES and DMEG have been observed (i.e., LR12-033). The cutaneous vascular malformations have a variable presentation from a classic port-wine stain appearance to pale pink or red capillary malformations. Body overgrowth including macrodactyly, lipomas, and lymphatic malformations are segmental and often severe with rapid postnatal progression. Epidermal nevi are common (19, 41).

Our data also show that the mutational spectrum of these disorders is different. The mutations associated with MCAP syndrome in particular are distinct from other *PIK3CA*-related overgrowth disorders. The mutational spectrum in MCAP is broad, with greater than 20 milder GOF mutations seen across the entire gene, whereas most mutations seen in CLOVES, KTS, DMEG, and other focal forms of overgrowth (such as isolated macrodactyly) are more often associated with the cancer hotspot mutations. Given this wide mutational spectrum, we recommend using a broad molecular strategy that tests the entire *PIK3CA* coding sequence, rather than targeted mutation screening of common mutations in individuals with similar phenotypes. Several individuals lacked megalencephaly, and had variable somatic overgrowth with vascular malformations, further expanding the spectrum of *PIK3CA*-related overgrowth. Therefore, the molecular data on *PIK3CA*-related overgrowth disorders to date show that they comprise a discontinuous spectrum of disorders that correlate well with the severity (strength of GOF) and distribution of the mutation, rather than a single continuous spectrum.

### Diagnosis of mosaicism in developmental disorders

Genetic disorders caused by postzygotic (or mosaic) variants are increasingly recognized with the advent of deep NGS methods. The contribution of mosaic variation to developmental brain disorders has also been increasingly appreciated in various segmental disorders including HMEG, FCD, and, more recently, bilateral perisylvian polymicrogyria (16, 20, 27, 36, 48–51). However, detecting low-frequency mosaic variants in clinical practice remains challenging for several reasons. First, the availability of nonblood tissue sources (such as affected skin or brain) may be limited. Second, available diagnostic testing methods used in clinical diagnostic labs (such as Sanger sequencing or standard-depth NGS) are insufficiently sensitive to detect mosaic variation. We therefore sought to address these issues by sequencing large cohorts of samples from individuals with overgrowth in general, and MCAP in particular, using 2 independent deep-targeted NGS methods that utilize MIPs and amplicon deep sequencing. These methods have been particularly demonstrated to be effective for detecting low-frequency variants (14, 15). When combined with single-molecule tagging with multiplex targeted sequencing (smMIPs), the MIP method further increases the sensitivity for the detection of low-frequency variants, as single-molecule tagging marks sequences derived from a common progenitor molecule. Our results also challenge the current clinical practice of using blood-derived DNA for almost all clinical genetic testing. Our study suggests that a change in clinical practice is indicated for disorders suspected to be associated with somatic mosaicism, such as those involving asymmetry or other segmental or focal presentation, i.e., as a large majority of vascular malformations, overgrowth, cutaneous pigmentary abnormalities, noncancerous dysplasia (i.e., epidermal nevi).

### Summary

Our data expand on the molecular and phenotypic spectrum of *PIK3CA*-related developmental disorders. First, we show that the mutational spectrum in children with MCAP is broader than other *PIK3CA*-related overgrowth disorders. Further, we report on several atypical or expanded phenotypes, including children with body overgrowth without megalencephaly, that appear milder than CLOVES and other severe forms of somatic overgrowth. Finally, we report on constitutional mutations of *PIK3CA* in a subset of children, some of whom have milder features such as diffuse megalencephaly with intellectual disability, similar to the *PTEN*-related disorders (52). Based on our data and review of the literature, we propose a molecularly based classification of these disorders. Overall, our molecular studies in this large series show that phenotypes of mosaic disorders are impacted not only by tissue distribution and levels of mosaicism, but also by class (strength of activation) of mutation. Our series helps inform best clinical practices for diagnostic methods and tissues to sample in mosaic disorders broadly.

## Methods

### Sample processing.

Genomic DNA was extracted from blood using the Puregene Blood Core Kit (QIAGEN), saliva using the Oragene Saliva Kit (DNA Genotek), buccal cells from Oragene Saliva Kits following centrifugation, and skin fibroblasts following a skin biopsy. Fibroblasts were grown in DMEM/F12 with glutamine, supplemented with 10% FBS and 1% PenStrep (all three from Gibco). DNA was extracted both directly and from fibroblasts after digesting with proteinase K in Cell Lysis Buffer (both QIAGEN) using prepIT.L2P (DNA Genotek) and ethanol precipitation. Lymphocytes were separated from whole blood using Ficoll and subsequently transformed to lymphoblastoid (LB) cell lines using Epstein-Barr virus (EBV) in RPMI 1640 media with glutamine, supplemented with 10% FBS and 1% PenStrep (all three from Gibco). Genomic DNA was then extracted using a Puregene Blood Core Kit.

### Multiplex targeted sequencing using smMIPs.

We designed a pool of 48 smMIP oligonucleotide probes targeting the coding sequences of *PIK3CA*. smMIPs tiled across a total of 3,340 bp of genomic sequence, including 100% of the 2,202 coding bp of *PIK3CA*. One hundred–nanogram capture reactions were performed in parallel. Massively parallel sequencing was performed with the Illumina HiSeq. Variant analysis was performed using our previously published pipeline including MIPgen and PEAR version 0.8.1 (48, 53). All missense, nonsense, and splice site variants seen at a frequency of less than 1% in public databases, in 2 or more capture events were retained for analysis. Our MIP capture method has been previously published (48).

### Amplicon sequencing.

Amplicon sequencing was performed using previously published methods (48). We performed locus-specific amplification of genomic DNA followed by GS Junior sequencing (Roche). We designed fusion primers containing genome-specific sequences along with distinct multiplex identifier sequences (used to differentiate samples being run together on the same plate) and sequencing adapters, to generate amplicons ranging in size from 290 to 310 bp, using Primer3Plus software. Primer sequences are provided in Supplemental Table 4. Small DNA fragments were removed with Agencourt AMPure XP (Beckman Coulter) according to the manufacturer’s protocol. All amplicons were quantified with the Quant-iT PicoGreen dsDNA reagent (Life Technologies), pooled at equimolar ratios, amplified by emulsion PCR using the GS Junior Titanium emPCR kit (Lib-A kit, Roche Applied Science), and pyrosequenced in the sense and antisense strands on a GS Junior sequencer following the manufacturers’ instructions. Data were analyzed using GS Amplicon Variant Analyzer version 3.0 software.

### WES.

WES produced 18.2 GB of sequencing data per proband. Mean coverage of captured regions was ~250× for proband samples, with ~99.17% covered with at least 10× coverage, an average of 89.82% of base call quality of Q30 or greater, and an overall average mean quality score of Q35. WES was performed using previously published methods (54).

### Targeted NGS.

This assay sequenced all exons of *PIK3CA*, and average coverage ranged from 320 to greater than 1,000 sequencing reads per bp. Genomic regions were captured using biotinylated RNA oligonucleotides (SureSelect), prepared in paired-end libraries with ~200-bp insert size, and sequenced on an Illumina HiSeq2500 instrument with 100-bp read lengths, in a modification of a previously reported pipeline (55). Large deletions and duplications were detected by this panel using previously published methods (56).

### Statistics.

We completed a pairwise comparison of mean values for alternative allele percentages in the three tissue types, where a 2-tailed *t* test gave *P* values of 0.0352, 0.0365, and 0.566 for blood versus saliva, blood versus skin, and saliva versus skin, respectively. A *P* value less than 0.05 was considered statistically significant.

### Study approval.

This study was approved by the IRB at Seattle Children’s Hospital. Informed consent was obtained from subjects prior to enrollment in the study, except for subjects whose samples and clinical data were sent without identifying information. De-identified subjects were included under IRB waiver of consent, per IRB protocol at Seattle Children’s Hospital. Written informed consent was provided for use of the patients’ photographs in the clinical figures of this manuscript.

## Author contributions

GMM designed the research study, conducted experiments, analyzed data, and wrote the manuscript. AET and AG analyzed data and assisted in writing the manuscript. VC, EAB, MK, SC, and BM conducted experiments and assisted with data analysis. CO and KP recruited subjects to this study and assisted with acquiring data. JJ, CP, and KT assisted with data acquisition and analysis. MC, IG, GKM, IKM, DC, NS, ASP, RB, ET, SB, LB, SEC, SG, MS, FS, CP, MN, SS, RS, MG, BOC, CEK, LJY, AHM, MM, MCA, LB, RC, KD, KTB, MEP, MP, LW, JR, RH, MA, KG, MC, HvE, LG, CC, HK, and JG assisted with acquiring data. JS and RG helped design the study. WBD designed the research study and wrote the manuscript.

## Supplementary Material

Supplemental data

## Figures and Tables

**Table 5:**
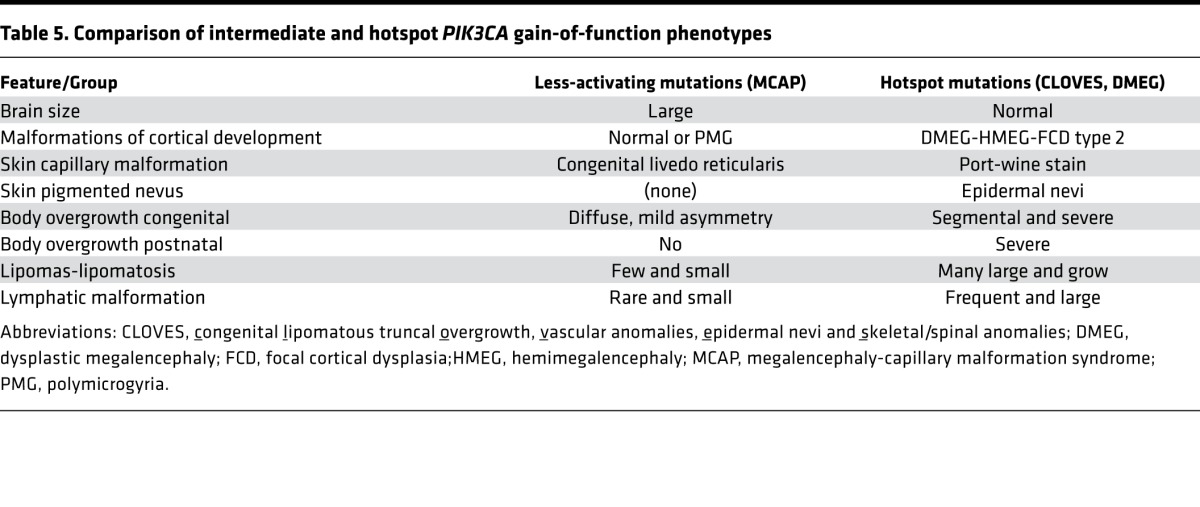
Comparison of intermediate and hotspot *PIK3CA* gain-of-function phenotypes

**Table 4:**
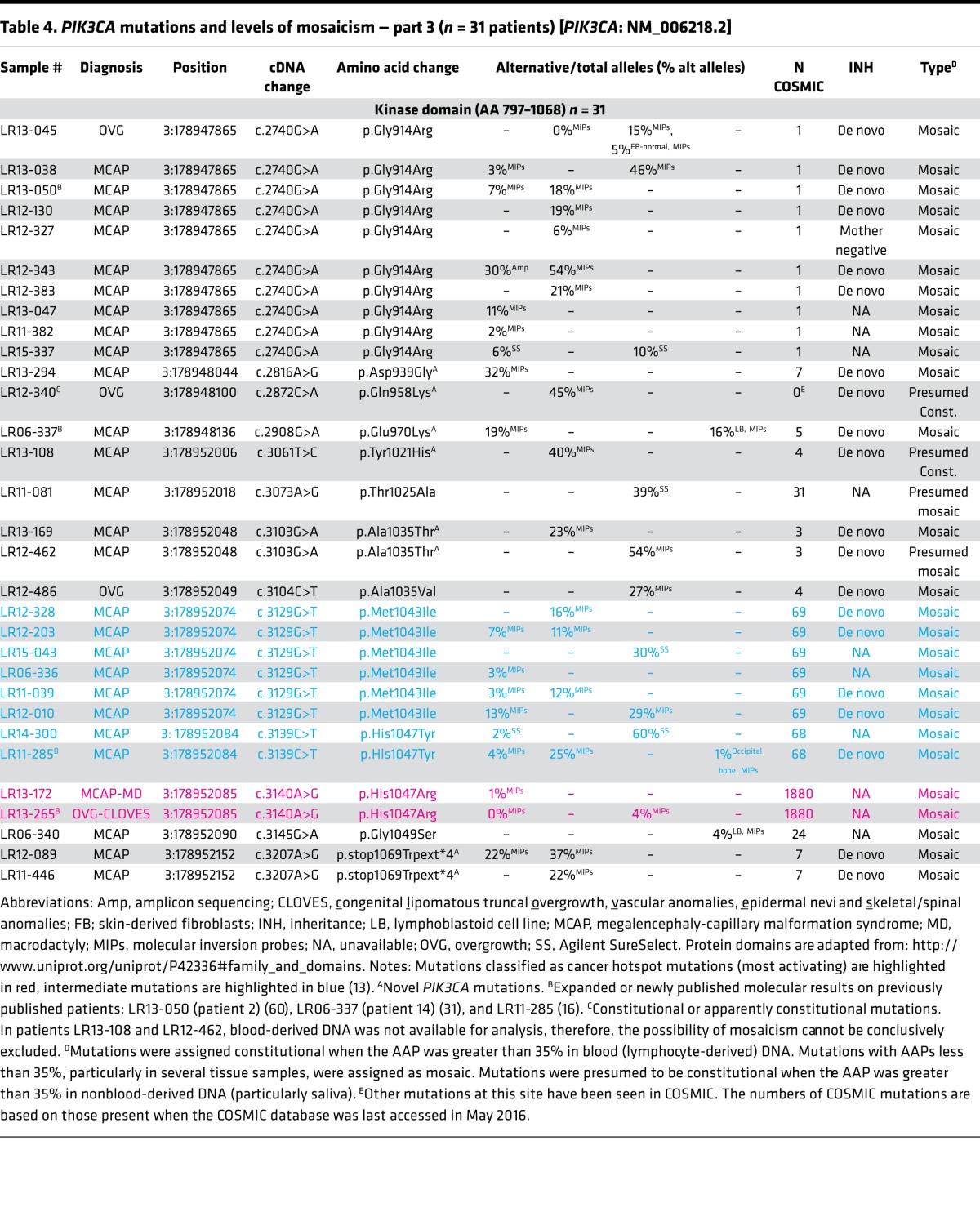
*PIK3CA* mutations and levels of mosaicism — part 3 (*n* = 31 patients) [*PIK3CA*: NM_006218.2]

**Table 3:**
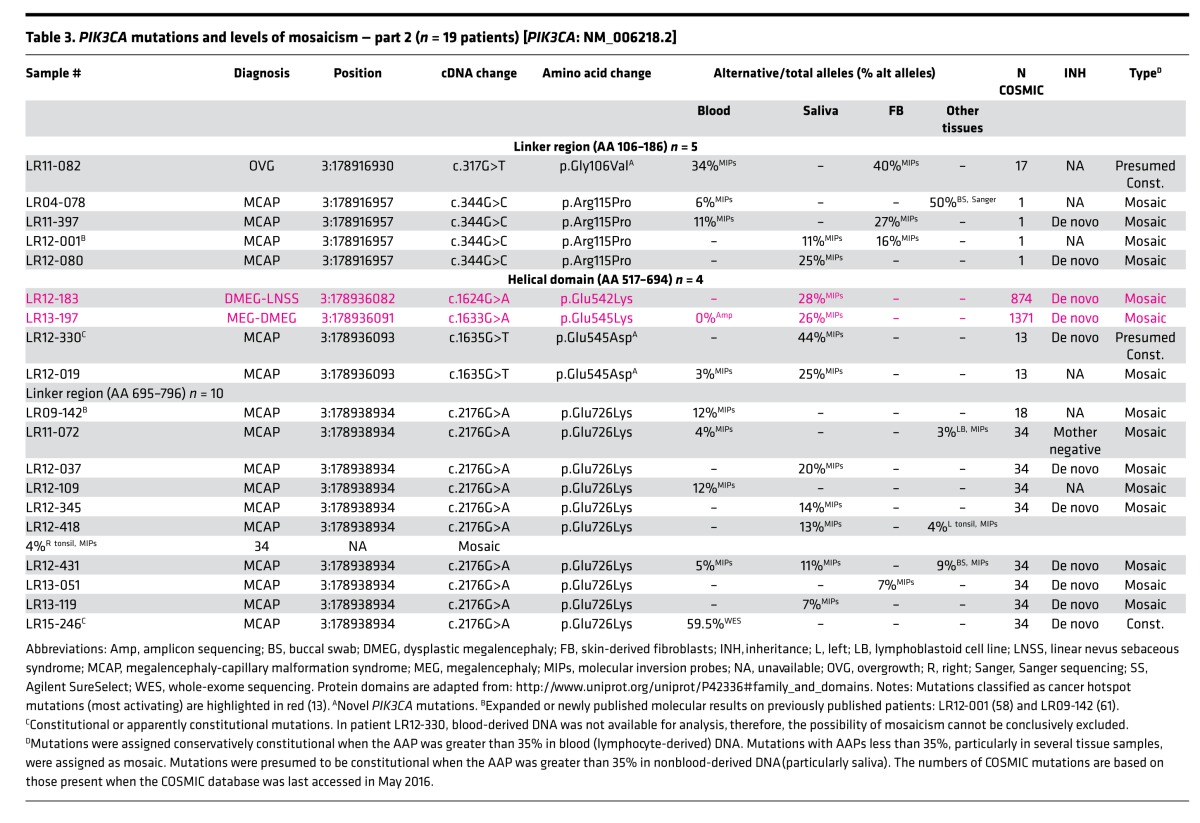
*PIK3CA* mutations and levels of mosaicism — part 2 (*n* = 19 patients) [*PIK3CA*: NM_006218.2]

**Table 2:**
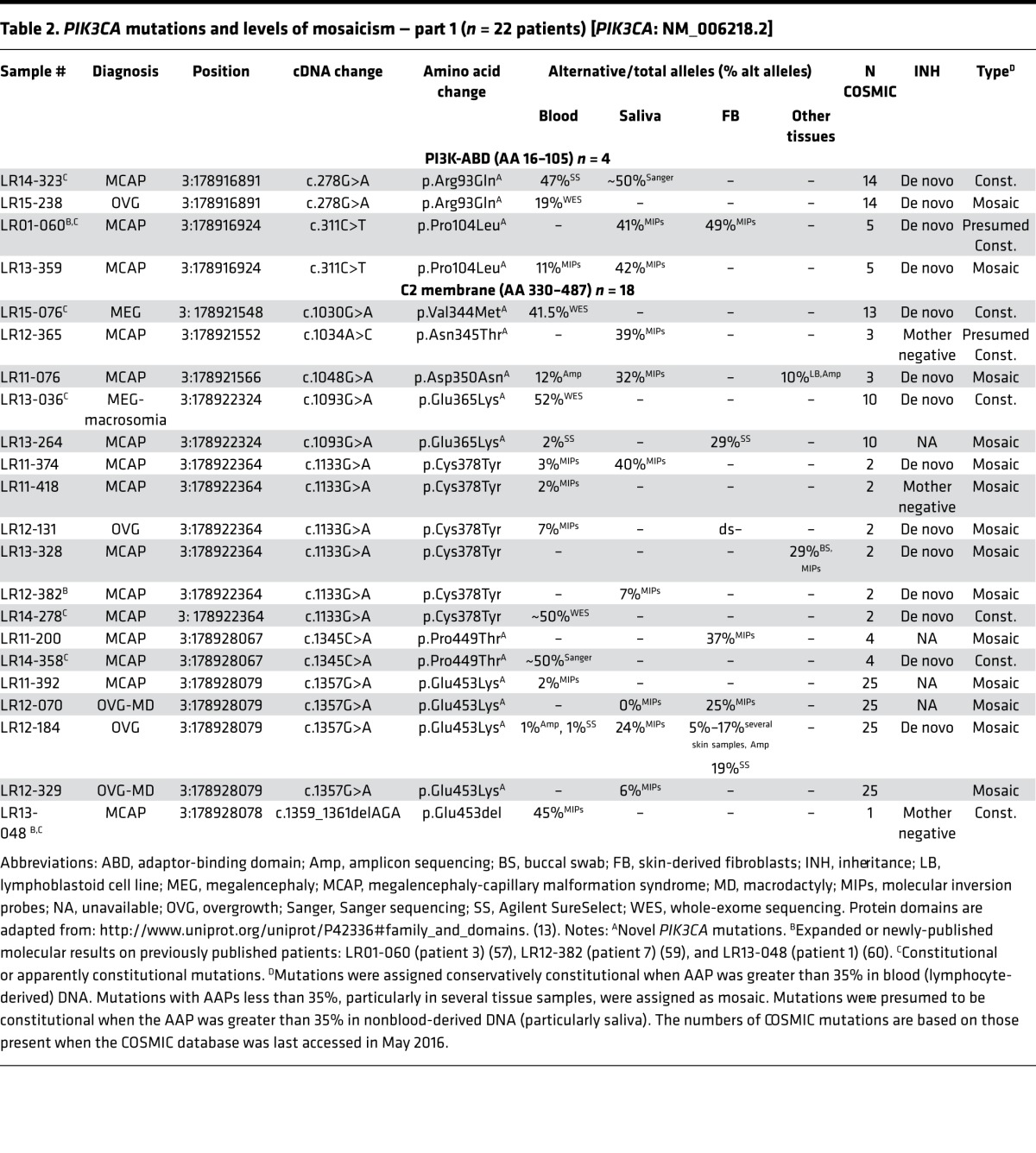
*PIK3CA* mutations and levels of mosaicism — part 1 (*n* = 22 patients) [*PIK3CA*: NM_006218.2]

**Table 1:**
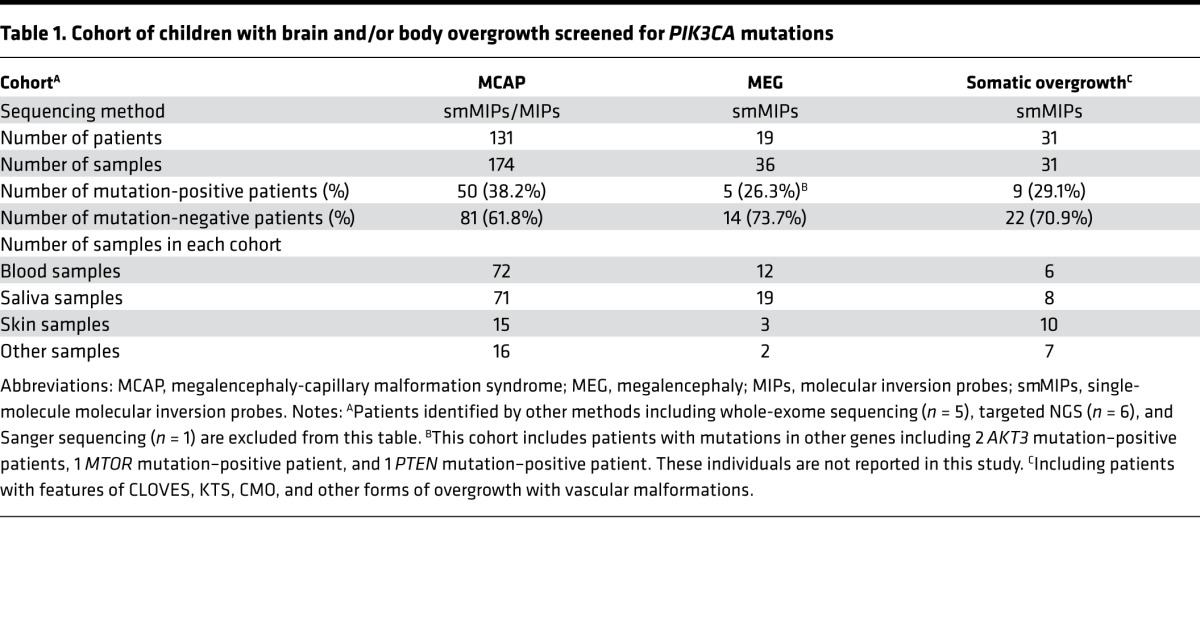
Cohort of children with brain and/or body overgrowth screened for *PIK3CA* mutations

## References

[1] Vattathil S, Scheet P (2016). Extensive hidden genomic mosaicism revealed in normal tissue. Am J Hum Genet.

[2] Biesecker LG, Spinner NB (2013). A genomic view of mosaicism and human disease. Nat Rev Genet.

[3] Zellweger H, Abbo G (1963). Chromosomal mosaicism and mongolism. Lancet.

[4] Pagon RA, Hall JG, Davenport SL, Aase J, Norwood TH, Hoehn HW (1979). Abnormal skin fibroblast cytogenetics in four dysmorphic patients with normal lymphocyte chromosomes. Am J Hum Genet.

[5] Weinstein LS, Shenker A, Gejman PV, Merino MJ, Friedman E, Spiegel AM (1991). Activating mutations of the stimulatory G protein in the McCune-Albright syndrome. N Engl J Med.

[6] Schwindinger WF, Francomano CA, Levine MA (1992). Identification of a mutation in the gene encoding the alpha subunit of the stimulatory G protein of adenylyl cyclase in McCune-Albright syndrome. Proc Natl Acad Sci USA.

[7] Happle R (1987). Lethal genes surviving by mosaicism: a possible explanation for sporadic birth defects involving the skin. J Am Acad Dermatol.

[8] Hall JG (1988). Review and hypotheses: somatic mosaicism: observations related to clinical genetics. Am J Hum Genet.

[9] Rehm HL (2013). ACMG clinical laboratory standards for next-generation sequencing. Genet Med.

[10] Keppler-Noreuil KM (2015). PIK3CA-related overgrowth spectrum (PROS): diagnostic and testing eligibility criteria, differential diagnosis, and evaluation. Am J Med Genet A.

[11] Engelman JA, Luo J, Cantley LC (2006). The evolution of phosphatidylinositol 3-kinases as regulators of growth and metabolism. Nat Rev Genet.

[12] Samuels Y, Ericson K (2006). Oncogenic PI3K and its role in cancer. Curr Opin Oncol.

[13] Gymnopoulos M, Elsliger MA, Vogt PK (2007). Rare cancer-specific mutations in PIK3CA show gain of function. Proc Natl Acad Sci U S A.

[14] Hiatt JB, Pritchard CC, Salipante SJ, O’Roak BJ, Shendure J (2013). Single molecule molecular inversion probes for targeted, high-accuracy detection of low-frequency variation. Genome Res.

[15] O’Roak BJ (2012). Multiplex targeted sequencing identifies recurrently mutated genes in autism spectrum disorders. Science.

[16] Rivière JB (2012). De novo germline and postzygotic mutations in AKT3, PIK3R2 and PIK3CA cause a spectrum of related megalencephaly syndromes. Nat Genet.

[17] Hafner C (2007). Oncogenic PIK3CA mutations occur in epidermal nevi and seborrheic keratoses with a characteristic mutation pattern. Proc Natl Acad Sci U S A.

[18] Groesser L, Herschberger E, Landthaler M, Hafner C (2012). FGFR3, PIK3CA and RAS mutations in benign lichenoid keratosis. Br J Dermatol.

[19] Kurek KC (2012). Somatic mosaic activating mutations in PIK3CA cause CLOVES syndrome. Am J Hum Genet.

[20] Lee JH (2012). De novo somatic mutations in components of the PI3K-AKT3-mTOR pathway cause hemimegalencephaly. Nat Genet.

[21] Lindhurst MJ (2012). Mosaic overgrowth with fibroadipose hyperplasia is caused by somatic activating mutations in PIK3CA. Nat Genet.

[22] Rios JJ (2013). Somatic gain-of-function mutations in PIK3CA in patients with macrodactyly. Hum Mol Genet.

[23] Cohen AS (2014). Somatic mosaicism for the p.His1047Arg mutation in PIK3CA in a girl with mesenteric lipomatosis. Am J Med Genet A.

[24] Keppler-Noreuil KM (2014). Clinical delineation and natural history of the PIK3CA-related overgrowth spectrum. Am J Med Genet A.

[25] Maclellan RA (2014). PIK3CA activating mutations in facial infiltrating lipomatosis. Plast Reconstr Surg.

[26] Loconte DC (2015). Molecular and functional characterization of three different postzygotic mutations in PIK3CA-related overgrowth spectrum (PROS) patients: effects on PI3K/AKT/mTOR signaling and sensitivity to PIK3 inhibitors. PLoS ONE.

[27] D’Gama AM (2015). Mammalian target of rapamycin pathway mutations cause hemimegalencephaly and focal cortical dysplasia. Ann Neurol.

[28] Luks VL (2015). Lymphatic and other vascular malformative/overgrowth disorders are caused by somatic mutations in PIK3CA. J Pediatr.

[29] Limaye N (2015). Somatic activating PIK3CA mutations cause venous malformation. Am J Hum Genet.

[30] Orloff MS (2013). Germline PIK3CA and AKT1 mutations in Cowden and Cowden-like syndromes. Am J Hum Genet.

[31] Conway RL (2007). Neuroimaging findings in macrocephaly-capillary malformation: a longitudinal study of 17 patients. Am J Med Genet A.

[32] Mirzaa GM (2012). Megalencephaly-capillary malformation (MCAP) and megalencephaly-polydactyly-polymicrogyria-hydrocephalus (MPPH) syndromes: two closely related disorders of brain overgrowth and abnormal brain and body morphogenesis. Am J Med Genet A.

[33] Gellis SS, Feingold M (1970). Linear nevus sebaceous syndrome. Am J Dis Child.

[34] Dobyns WB, Garg BP (1991). Vascular abnormalities in epidermal nevus syndrome. Neurology.

[35] Dodge NN, Dobyns WB (1995). Agenesis of the corpus callosum and Dandy-Walker malformation associated with hemimegalencephaly in the sebaceous nevus syndrome. Am J Med Genet.

[36] Jansen LA (2015). PI3K/AKT pathway mutations cause a spectrum of brain malformations from megalencephaly to focal cortical dysplasia. Brain.

[37] Klippel M, Trenaunay P (1900). Du naevus variqueux osteo-hypertrophique. Arch Gen Med.

[38] Happle R (2016). The categories of cutaneous mosaicism: a proposed classification. Am J Med Genet A.

[39] Vahidnezhad H, Youssefian L, Uitto J (2016). Klippel-Trenaunay syndrome belongs to the PIK3CA-related overgrowth spectrum (PROS). Exp Dermatol.

[40] Sapp JC, Turner JT, van de Kamp JM, van Dijk FS, Lowry RB, Biesecker LG (2007). Newly delineated syndrome of congenital lipomatous overgrowth, vascular malformations, and epidermal nevi (CLOVE syndrome) in seven patients. Am J Med Genet A.

[41] Alomari AI (2009). Characterization of a distinct syndrome that associates complex truncal overgrowth, vascular, and acral anomalies: a descriptive study of 18 cases of CLOVES syndrome. Clin Dysmorphol.

[42] Lee MS, Liang MG, Mulliken JB (2013). Diffuse capillary malformation with overgrowth: a clinical subtype of vascular anomalies with hypertrophy. J Am Acad Dermatol.

[43] Oduber CE (2011). A proposal for classification of entities combining vascular malformations and deregulated growth. Eur J Med Genet.

[44] Moore CA (1997). Macrocephaly-cutis marmorata telangiectatica congenita syndrome: a distinct disorder with developmental delay and connective tissue abnormality. Am J Med Genet.

[45] Clayton-Smith J (1997). Macrocephaly with cutis marmorata, haemangioma and syndactyly — a distinctive overgrowth syndrome. Clin Dysmorphol.

[46] Couto JA (2015). Facial infiltrating lipomatosis contains somatic PIK3CA mutations in multiple tissues. Plast Reconstr Surg.

[47] Di Donato N (2016). Identification and characterization of a novel constitutional PIK3CA mutation in a child lacking the typical segmental overgrowth of “PIK3CA-related overgrowth spectrum.”. Hum Mutat.

[48] Mirzaa GM (2015). Characterisation of mutations of the phosphoinositide-3-kinase regulatory subunit, PIK3R2, in perisylvian polymicrogyria: a next-generation sequencing study. Lancet Neurol.

[49] Leventer RJ (2015). Hemispheric cortical dysplasia secondary to a mosaic somatic mutation in MTOR. Neurology.

[50] Lim JS (2015). Brain somatic mutations in MTOR cause focal cortical dysplasia type II leading to intractable epilepsy. Nat Med.

[51] Nakashima M (2015). Somatic Mutations in the MTOR gene cause focal cortical dysplasia type IIb. Ann Neurol.

[52] Eng C (2003). PTEN: one gene, many syndromes. Hum Mutat.

[53] Boyle EA, O’Roak BJ, Martin BK, Kumar A, Shendure J (2014). MIPgen: optimized modeling and design of molecular inversion probes for targeted resequencing. Bioinformatics.

[54] Tanaka AJ (2015). Mutations in SPATA5 are associated with microcephaly, intellectual disability, seizures, and hearing loss. Am J Hum Genet.

[55] Pritchard CC (2012). ColoSeq provides comprehensive lynch and polyposis syndrome mutational analysis using massively parallel sequencing. J Mol Diagn.

[56] Nord AS, Lee M, King MC, Walsh T (2011). Accurate and exact CNV identification from targeted high-throughput sequence data. BMC Genomics.

[57] Mirzaa G (2004). Megalencephaly and perisylvian polymicrogyria with postaxial polydactyly and hydrocephalus: a rare brain malformation syndrome associated with mental retardation and seizures. Neuropediatrics.

[58] Swarr DT (2013). Expanding the differential diagnosis of fetal hydrops: an unusual prenatal presentation of megalencephaly-capillary malformation syndrome. Prenat Diagn.

[59] Stephan MJ, Hall BD, Smith DW, Cohen MM (1975). Macrocephaly in association with unusual cutaneous angiomatosis. J Pediatr.

[60] Vogels A (1998). The macrocephaly-cutis marmorata telangiectatica congenita syndrome. Long-term follow-up data in 4 children and adolescents. Genet Couns.

[61] McDermott JH, Byers H, Clayton-Smith J (2016). Detection of a mosaic PIK3CA mutation in dental DNA from a child with megalencephaly capillary malformation syndrome. Clin Dysmorphol.

[62] Saul RA, Schwartz CE, Stevenson R (1990). DNA evidence for somatic mosaicism in monozygotic twins discordant for proteus syndrome. Proceedings of the Greenwood Genetic Center.

