# Supplementary material for: PIK3CA-associated developmental disorders exhibit distinct classes of mutations with variable expression and tissue distribution
Source: JCI Insight. 2016 Jun 16;1(9):e87623. doi: 10.1172/jci.insight.87623 (PMC5019182; doi:10.1172/jci.insight.87623)
Supplement: Supplemental data [file jciinsight-1-87623-s001.pdf]

Supplementary Appendix

Content	Title	Page numbers
Supplementary Figure 1	Cohorts and samples	2
Supplementary Figure 2	Levels of mosaicism in *PIK3CA* in the study cohort.	3
Supplementary Figure 3	Alternative allele percentages (AAP) in all samples tested by smMIPs.	3
Supplementary Figure 4	Comparison between alternative allele percentages detected by smMIPs and amplicon sequencing.	4
Supplementary Figure 5	Clinical photographs of patient LR12-033	4
Supplementary Table 1	Molecular data of all mutation positive patients	5-22
Supplementary Table 2	Summary of clinical data of *PIK3CA* mutation positive patients	23-29
Supplementary Table 3	Detailed clinical data of *PIK3CA* mutation positive patients	30-47
Supplementary Table 4	*PIK3CA* primer sequences used for amplicon sequencing	48

SUPPLEMENTARY FIGURES

Supplementary Figure 1. Cohorts and samples. Pie charts showing the cohort screened for *PIK3CA* mutations by MIPs including 88 children with the megalencephaly-capillary malformation syndrome (MCAP), 19 children with megalencephaly (MEG), 31 children with somatic overgrowth or vascular malformations (OVG-VASC). Types of samples are color coded (red = blood; blue = saliva; orange = skin fibroblasts and gray = other tissues). Patients tested by other methods, such as clinical whole exome sequencing, are not included in this figure.

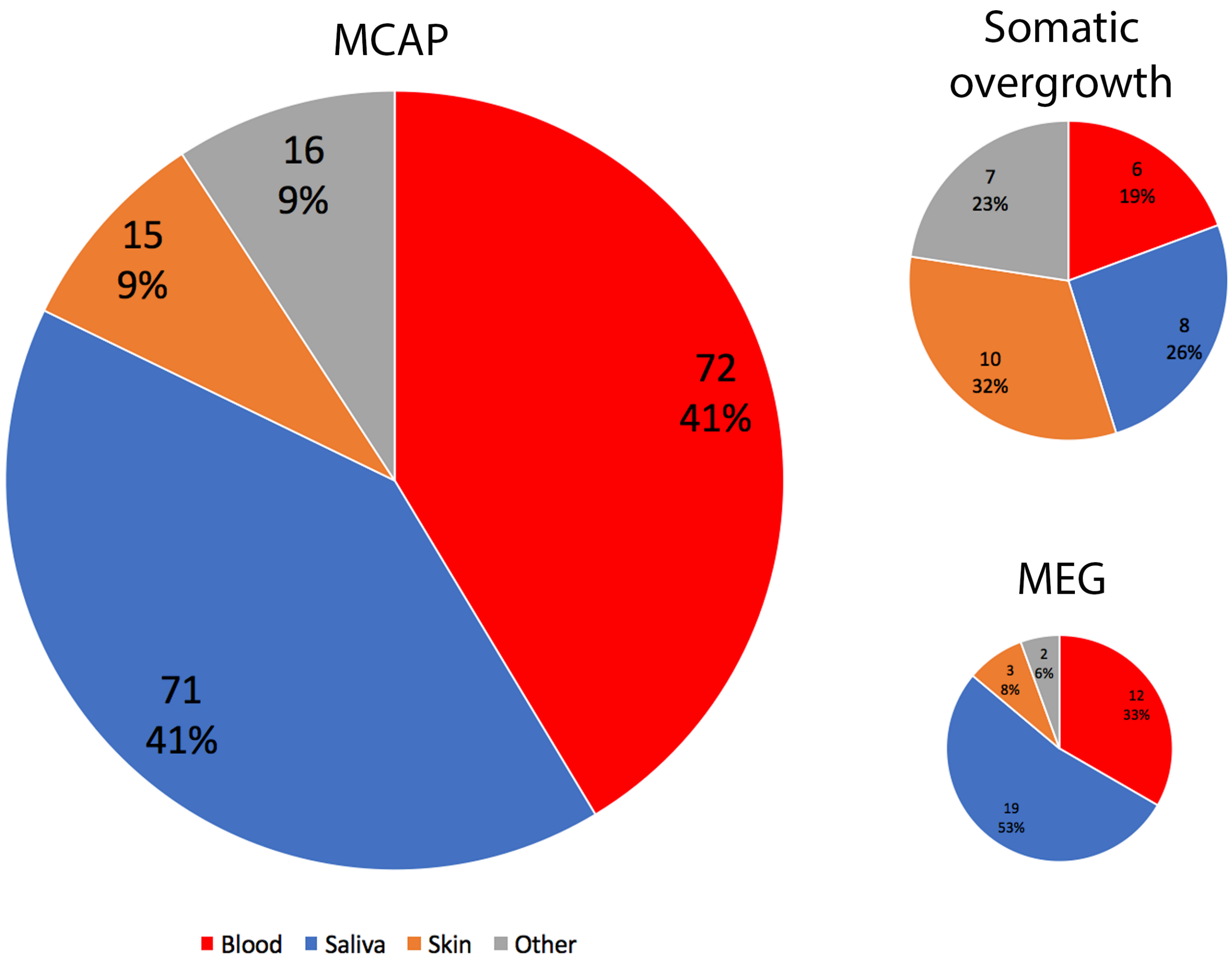

Supplementary Figure 2. Levels of mosaicism in *PIK3CA* in the study cohort. Dot blot graph showing alternative allele percentages (AAP) in different tissue types per individual. Each ID on the x-axis indicates a different individual with a confirmed *PIK3CA* mutation. Colors indicate different tissue types (red = blood; blue = saliva; orange = skin fibroblasts). Total number of samples = 108. ID = patient number.

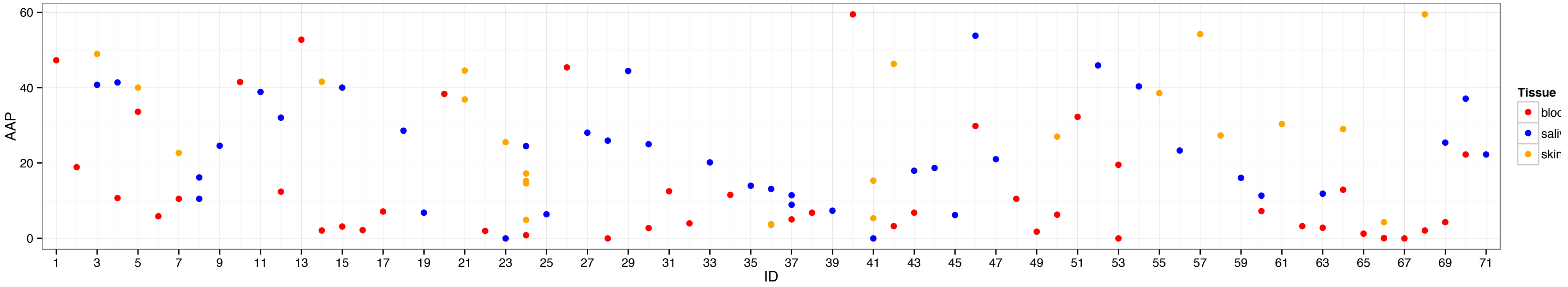

Supplementary Figure 3. Alternative allele percentages (AAP) in all samples (N=188) tested by smMIPs. Graph showing alternative allele frequency (AAP) in all samples tested by smMIPs for all positions where a mutation was identified. Red open circles indicate samples where mutations have been verified. Blue indicates samples where no mutations have been identified. Several samples showed spurious non-reference reads at amino acid residue p.Glu542 that appeared to be sequencing artifacts.

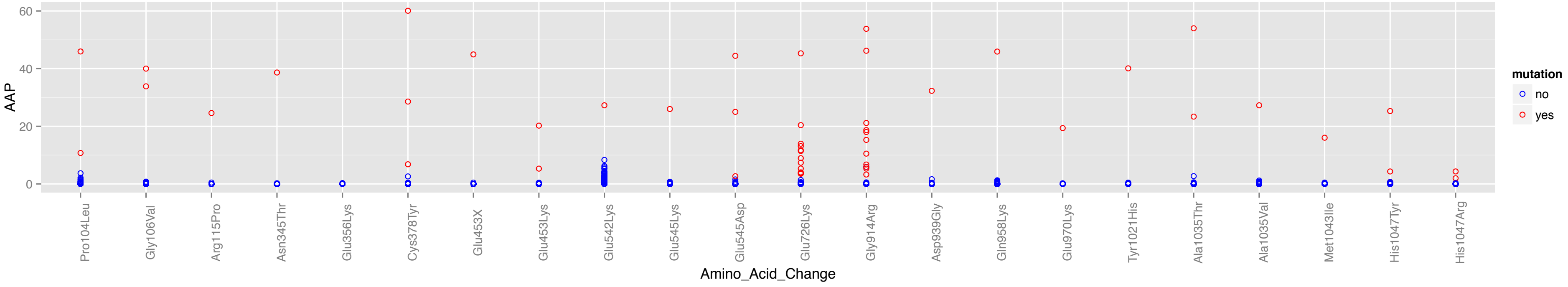

Supplementary Figure 4. Comparison between alternative allele percentages detected by smMIPs and amplicon sequencing. Graph comparing alternate allele frequencies detected by single molecule molecular inversion probes (smMIPs) and amplicon sequencing. For all samples analyzed by smMIPs and amplicon sequence AAP was graphed. Blue line is a linear regression line.

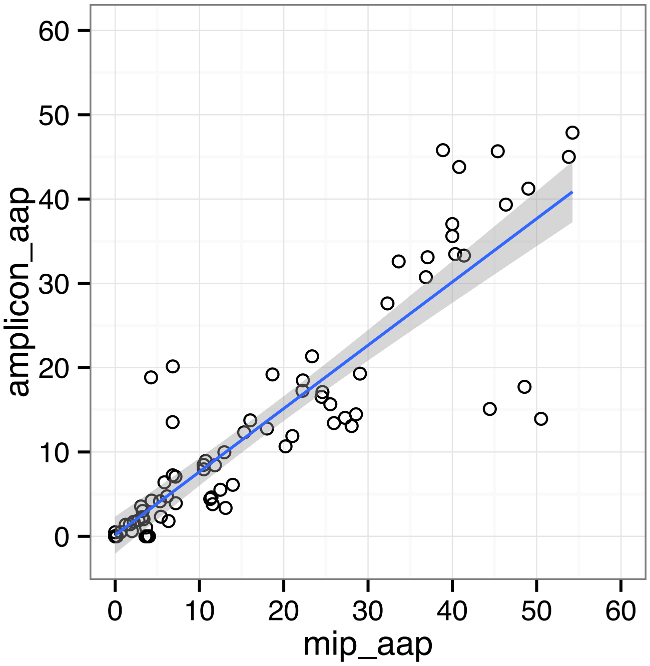

Supplementary Figure 5. Clinical photographs of patient LR12-033 (p.Glu545Lys). This previously published child has dysplastic megalencephaly and CLOVES syndrome features. A, facial photograph reveals apparent facial asymmetry; B, epidermal nevi of the skin; C, D, macrodactyly of both hands; E, severe asymmetric segmental overgrowth of the digits including macrodactyly of the toes.

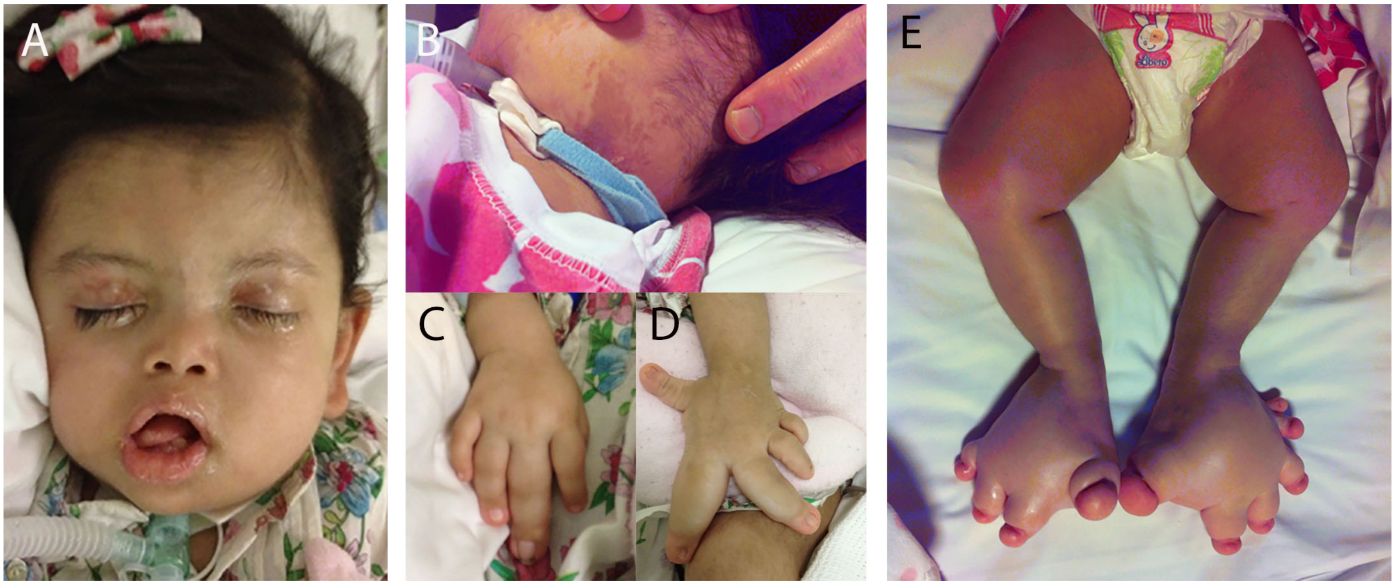

SUPPLEMENTARY TABLES

Supplementary Table 1. Mutations, tissue distribution and levels of mosaicism of *PIK3CA* mutation-positive patients (N = 72)

Database #	Tissue tested	Genomic Coordinates	cDNA change	Amino acid change	Method 1	Method 1	Method 2	Method 2
					Alternate/total alleles (AAP)	Method type	Alternate/total alleles (AAP)	Method type
**PI3K-ABD (AA 16-105) N = 4**								
**Family 1**								
LR14-323 (S1)	blood	3:178916891	c.278G>A	p.Arg93Gln	451/954 (47.3)	Agilent SS	–	–
LR14-323 (S2)	saliva	3:178916891	c.278G>A	p.Arg93Gln	NA (50.0)	Sanger SEQ	–	–
LR14-323f	blood	3:178916891	c.278G>A	p.Arg93Gln	0/496 (0)	Agilent SS	NA (0)	Sanger SEQ
LR14-323m	blood	3:178916891	c.278G>A	p.Arg93Gln	0/425 (0)	Agilent SS	NA (0)	Sanger SEQ
**Family 2**								
LR15-238	blood	3:178916891	c.278G>A	p.Arg93Gln	30/159 (19)	WES	–	–
LR15-238f	blood	3:178916891	c.278G>A	p.Arg93Gln	NA (0)	WES	–	–
LR15-238m	blood	3:178916891	c.278G>A	p.Arg93Gln	NA (0)	WES	–	–
**Family 3**								
LR01-060 (S1)	saliva	3:178916924	c.311C>T	p.Pro104Leu	111/272 (40.8)	Sanger SEQ	1577/3600 (43.8)	Amplicon SEQ
LR01-060 (S2)	skin FB	3:178916924	c.311C>T	p.Pro104Leu	100/204 (49.0)	Sanger SEQ	379/919 (41.2)	Amplicon SEQ
LR01-060f	saliva	3:178916924	c.311C>T	p.Pro104Leu	NA (0)	Sanger SEQ	0/2175 (0)	Amplicon SEQ
LR01-060m	saliva	3:178916924	c.311C>T	p.Pro104Leu	NA (0)	Sanger SEQ	0/1358 (0)	Amplicon SEQ

**Family 4**								
LR13-359 (S1)	blood	3:178916924	c.311C>T	p.Pro104Leu	13/121 (10.7)	smMIPs	303/3391 (8.9)	Amplicon SEQ
LR13-359 (S2)	saliva	3:178916924	c.311C>T	p.Pro104Leu	24/58 (41.4)	smMIPs	1284/3856 (33.3)	Amplicon SEQ
LR13-359f	blood	3:178916924	c.311C>T	p.Pro104Leu	0/13 (0)	Amplicon SEQ	–	–
LR13-359m	saliva	3:178916924	c.311C>T	p.Pro104Leu	0/4769 (0)	Amplicon SEQ	–	–
**Linker region (AA 106-186) N = 5**								
**Family 5**								
LR11-082 (S1)	skin FB	3:178916930	c.317G>T	p.Gly106Val	24/60 (40.0)	smMIPs	1313/3688 (35.6)	Amplicon SEQ
LR11-082 (S2)	blood	3:178916930	c.317G>T	p.Gly106Val	150/446 (33.6)	smMIPs	1038/3184 (32.6)	Amplicon SEQ
**Family 6**								
LR04-078	blood	3:178916957	c.344G>C	p.Arg115Pro	28/479 (5.84)	smMIPs	230/3592 (6.4)	Amplicon SEQ
**Family 7**								
LR11-397 (S1)	blood	3:178916957	c.344G>C	p.Arg115Pro	59/562 (10.5)	smMIPs	1118/13194 (8.5)	Amplicon SEQ
LR11-397 (S2)	skin FB	3:178916957	c.344G>C	p.Arg115Pro	806/3559 (22.7)	Amplicon SEQ	–	–
LR11-397f	blood	3:178916957	c.344G>C	p.Arg115Pro	0/2289 (0)	Amplicon SEQ	–	–
LR11-397m	blood	3:178916957	c.344G>C	p.Arg115Pro	0/5614 (0)	Amplicon SEQ	–	–

**Family 8**								
LR12-001 (S1)	saliva	3:178916957	c.344G>C	p.Arg115Pro	22/210 (10.5)	smMIPs	–	–
LR12-001 (S2)	saliva	3:178916957	c.344G>C	p.Arg115Pro	114/706 (16.2)	smMIPs	–	–
**Family 9**								
LR12-080	saliva	3:178916957	c.344G>C	p.Arg115Pro	15/61 (24.6)	smMIPs	477/2788 (17.1)	Amplicon SEQ
LR12-080f	saliva	3:178916957	c.344G>C	p.Arg115Pro	0/1738 (0)	Amplicon SEQ	–	–
LR12-080m	saliva	3:178916957	c.344G>C	p.Arg115Pro	0/2680 (0)	Amplicon SEQ	–	–
**C2 membrane (AA 330-487) N = 18**								
**Family 10**								
LR15-076	blood	3:178921548	c.1030G>A	p.Val344Met	44/106 (41.5)	WES	–	–
LR15-076f	blood	3:178921548	c.1030G>A	p.Val344Met	ND (0)	Sanger SEQ	–	–
LR15-076m	blood	3:178921548	c.1030G>A	p.Val344Met	ND (0)	Sanger SEQ	–	–
**Family 11**								
LR12-365	saliva	3:178921552	c.1034A>C	p.Asn345Thr	168/432 (38.9)	smMIPs	1892/4131 (45.8)	Amplicon SEQ
LR12-365m	saliva	3:178921552	c.1034A>C	p.Asn345Thr	0/2724 (0)	Amplicon SEQ	ND (0)	Sanger SEQ
**Family 12**								
LR11-076 (S1)	blood	3:178921566	c.1048G>A	p.Asp350Asn	536/4313 (12.4)	Amplicon SEQ	–	–

LR11-076 (S2)	saliva	3:178921566	c.1048G>A	p.Asp350Asn	119/371 (32.1)	smMIPs	failed x3	Amplicon SEQ
LR11-076 (S3)	LB	3:178921566	c.1048G>A	p.Asp350Asn	171/1751 (9.8)	Amplicon SEQ	–	–
LR11-076f (S1)	blood	3:178921566	c.1048G>A	p.Asp350Asn	0/2214 (0)	Amplicon SEQ	–	–
LR11-076f (S2)	LB	3:178921566	c.1048G>A	p.Asp350Asn	0/1716 (0)	Amplicon SEQ	–	–
LR11-076m (S1)	blood	3:178921566	c.1048G>A	p.Asp350Asn	0/1822 (0)	Amplicon SEQ	–	–
LR11-076m (S2)	LB	3:178921566	c.1048G>A	p.Asp350Asn	0/2918 (0)	Amplicon SEQ	–	–
**Family 13**								
LR13-036	blood	3:178922324	c.1093G>A	p.Glu365Lys	47/89 (52.8)	WES	–	–
LR13-036f	blood	3:178922324	c.1093G>A	p.Glu365Lys	ND (0)	WES	–	–
LR13-036m	blood	3:178922324	c.1093G>A	p.Glu365Lys	ND (0)	WES	–	–
**Family 14**								
LR13-264	blood	3:178922324	c.1093G>A	p.Glu365Lys	18/886 (2.0)	Agilent SS	–	–
LR13-264	skin FB	3:178922324	c.1093G>A	p.Glu365Lys	259/623 (29.0)	Agilent SS	–	–
**Family 15**								
LR11-374 (S1)	blood	3:178922364	c.1133G>A	p.Cys378Tyr	22/715 (3.1)	smMIPs	79/2230 (3.5)	Amplicon SEQ
LR11-374 (S2)	saliva	3:178922364	c.1133G>A	p.Cys378Tyr	86/215 (40.0)	smMIPs	660/1782 (37)	Amplicon SEQ
LR11-374f	saliva	3:178922364	c.1133G>A	p.Cys378Tyr	0/2241 (0)	Amplicon SEQ	–	–
LR11-374m	saliva	3:178922364	c.1133G>A	p.Cys378Tyr	0/2338 (0)	Amplicon SEQ	–	–
**Family 16**								
LR11-418	blood	3:178922364	c.1133G>A	p.Cys378Tyr	25/1136 (2.2)	smMIPs	23/1337 (1.7)	Amplicon SEQ
LR11-418f	blood	3:178922364	c.1133G>A	p.Cys378Tyr	0/1027 (0)	Amplicon SEQ	–	–

LR11-418m (S1)	saliva	3:178922364	c.1133G>A	p.Cys378Tyr	0/2097 (0)	Amplicon SEQ	–	–
LR11-418m (S2)	blood	3:178922364	c.1133G>A	p.Cys378Tyr	0/192 (0)	Amplicon SEQ	–	–
**Family 17**								
LR12-131	blood	3:178922364	c.1133G>A	p.Cys378Tyr	72/1006 (7.2)	smMIPs	230/3249 (7.1)	Amplicon SEQ
LR12-131f	saliva	3:178922364	c.1133G>A	p.Cys378Tyr	0/191 (0)	Amplicon SEQ	–	–
LR12-131m	saliva	3:178922364	c.1133G>A	p.Cys378Tyr	0/2389 (0)	Amplicon SEQ	–	–
**Family 18**								
LR13-328	buccal swab	3:178922364	c.1133G>A	c.Cys378Tyr	2/7 (28.6)	smMIPs	89/615 (14.5)	Amplicon SEQ
LR13-328f	blood	3:178922364	c.1133G>A	c.Cys378Tyr	0/3730 (0)	Amplicon SEQ	–	–
LR13-328m	blood	3:178922364	c.1133G>A	c.Cys378Tyr	0/1687 (0)	Amplicon SEQ	–	–
**Family 19**								
LR12-382	saliva	3:178922364	c.1133G>A	p.Cys378Tyr	3/44 (6.8)	smMIPs	503/3714 (13.5)	Amplicon SEQ
LR12-382f	saliva	3:178922364	c.1133G>A	p.Cys378Tyr	0/3356 (0)	Amplicon SEQ	–	–
LR12-382m	saliva	3:178922364	c.1133G>A	p.Cys378Tyr	0/3621 (0)	Amplicon SEQ	–	–
**Family 20**								
LR14-278	blood	3:178922364	c.1133G>A	p.Cys378Tyr	ND (~50)	WES	–	–
LR14-278f	blood	3:178922364	c.1133G>A	p.Cys378Tyr	NA (0)	WES	–	–
LR14-278m	blood	3:178922364	c.1133G>A	p.Cys378Tyr	NA (0)	WES	–	–
**Family 21**								

LR11-200 (S1)	skin FB	3:178928067	c.1345C>A	p.Pro449Thr	87/236 (36.9)	smMIPs	1183/3849 (30.7)	Amplicon SEQ
LR11-200 (S2)	skin FB	3:178928067	c.1345C>A	p.Pro449Thr	1922/4308 (44.6)	Amplicon SEQ	–	–
**Family 22**								
LR14-358	blood	3:178928067	c.1345C>A	p.Pro449Thr	NA (50.0)	Sanger SEQ	–	–
LR14-358f	blood	3:178928067	c.1345C>A	p.Pro449Thr	NA (0)	Sanger SEQ	–	–
LR14-358m	blood	3:178928067	c.1345C>A	p.Pro449Thr	NA (0)	Sanger SEQ	–	–
**Family 23**								
LR11-392	blood	3:178928079	c.1357G>A	p.Glu453Lys	11/552 (2)	smMIPs	23/3727 (0.6)	Amplicon SEQ
**Family 24**								
LR12-070 (S1)	saliva	3:178928079	c.1357G>A	p.Glu453Lys	0/91 (0)*	smMIPs	8/21 (38.1)	Amplicon SEQ
LR12-070 (S2)	skin FB	3:178928079	c.1357G>A	p.Glu453Lys	363/1422 (25.5)	smMIPs	469/2994 (15.7)	Amplicon SEQ
**Family 25**								
LR12-184 (S1)	blood	3:178928079	c.1357G>A	p.Glu453Lys	23/2705 (0.9)	Amplicon SEQ	13/1030 (1.0)	Agilent SS
LR12-184 (S2)	saliva	3:178928079	c.1357G>A	p.Glu453Lys	12/49 (24.5)	smMIPs	451/2726 (16.5)	Amplicon SEQ
LR12-184 (S3)	skin FB#1	3:178928079	c.1357G>A	p.Glu453Lys	639/3721 (17.2)	Amplicon SEQ	171/910 (19)	Agilent SS

LR12-184 (S4)	skin FB#2	3:178928079	c.1357G>A	p.Glu453Lys	520/3566 (14.6)	Amplicon SEQ	–	–
LR12-184 (S5)	skin FB#3	3:178928079	c.1357G>A	p.Glu453Lys	104/2097 (5)	Amplicon SEQ	–	–
LR12-184 (S6)	skin FB#4	3:178928079	c.1357G>A	p.Glu453Lys	533/3513 (15.2)	Amplicon SEQ	–	–
LR12-184f	saliva	3:178928079	c.1357G>A	p.Glu453Lys	0/4697 (0)	Amplicon SEQ	–	–
LR12-184m	saliva	3:178928079	c.1357G>A	p.Glu453Lys	0/4096 (0)	Amplicon SEQ	–	–
**Family 26**								
LR12-329	saliva	3:178928079	c.1357G>A	p.Glu453Lys	19/300 (6.3)	smMIPs	86/4758 (1.8)	Amplicon SEQ
LR12-329f (S1)	saliva	3:178928079	c.1357G>A	p.Glu453Lys	227/1802 (12.6)	Amplicon SEQ	–	–
LR12-329f (S2)	blood	3:178928079	c.1357G>A	p.Glu453Lys	0/4235 (0)	Amplicon SEQ	–	–
LR12-329m	saliva	3:178928079	c.1357G>A	p.Glu453Lys	0/4229 (0)	Amplicon SEQ	–	–
**Family 27**								
LR13-048	blood	3:178928078	c.1359_1361delAGA	p.Glu453X	251/553 (45.4)	smMIPs	854/1870 (45.7)	Amplicon SEQ
LR13-048m	blood	3:178928078	c.1359_1361delAGA	p.Glu453X	0/3275 (0)	Amplicon SEQ	–	–
**Helical domain (AA 517-694) N = 4**								
**Family 28**								
LR12-183	saliva	3:178936082	c.1624G>A	p.Glu542Lys	39/139 (28.1)	smMIPs	87/665 (13.1)	Amplicon SEQ
LR12-183f	saliva	3:178936082	c.1624G>A	p.Glu542Lys	0.655 (0)	Amplicon SEQ	–	–

LR12-183m	saliva	3:178936082	c.1624G>A	p.Glu542Lys	0/780 (0)	Amplicon SEQ	–	–
**Family 29**								
LR13-197 (S1)	blood	3:178936091	c.1633G>A	p.Glu545Lys	0/648 (0)	Amplicon SEQ	–	–
LR13-197 (S2)	saliva	3:178936091	c.1633G>A	p.Glu545Lys	150/578 (26)	smMIPs	96/715 (13.4)	Amplicon SEQ
LR13-197f	saliva	3:178936091	c.1633G>A	p.Glu545Lys	0/704 (0)	Amplicon SEQ	–	–
LR13-197m	saliva	3:178936091	c.1633G>A	p.Glu545Lys	0/831 (0)	Amplicon SEQ	–	–
**Family 30**								
LR12-330	saliva	3:178936093	c.1635G>T	p.Glu545Asp	20/45 (44.4)	smMIPs	119/788 (15.1)	Amplicon SEQ
LR12-330f	saliva	3:178936093	c.1635G>T	p.Glu545Asp	0/605 (0)	Amplicon SEQ	–	–
LR12-330m	saliva	3:178936093	c.1635G>T	p.Glu545Asp	0/624 (0)	Amplicon SEQ	–	–
**Family 31**								
LR12-019 (S1)	blood	3:178936093	c.1635G>T	p.Glu545Asp	7/263 (0.03)	smMIPs	–	–
LR12-019 (S2)	saliva	3:178936093	c.1635G>T	p.Glu545Asp	4/16 (0.25)	smMIPs	–	–
**Linker region (AA 695-796) N = 10**								
**Family 32**								
LR09-142	blood	3:178938934	c.2333G>A	p.Glu726Lys	161/1290 (12.5)	smMIPs	69/1250 (5.5)	Amplicon SEQ
**Family 33**								
LR11-072 (S1)	blood	3:178938934	c.2333G>A	p.Glu726Lys	12/299 (4)	smMIPs	0/330 (0)	Amplicon SEQ
LR11-072 (S2)	LB	3:178938934	c.2333G>A	p.Glu726Lys	76/2259 (3.4)	smMIPs	10/493 (2)	Amplicon SEQ

LR11-072m (S1)	LB	3:178938934	c.2333G>A	p.Glu726Lys	0/61 (0)	Amplicon SEQ	–	–
LR11-072m (S2)	blood	3:178938934	c.2333G>A	p.Glu726Lys	0/215 (0)	Amplicon SEQ	–	–
**Family 34**								
LR12-037	saliva	3:178938934	c.2333G>A	p.Glu726Lys	113/559 (20.2)	smMIPs	8/75 (10.7)	Amplicon SEQ
LR12-037f	saliva	3:178938934	c.2333G>A	p.Glu726Lys	0/662 (0)	Amplicon SEQ	–	–
LR12-037m	saliva	3:178938934	c.2333G>A	p.Glu726Lys	0/658 (0)	Amplicon SEQ	–	–
**Family 35**								
LR12-109	blood	3:178938934	c.2333G>A	p.Glu726Lys	82/709 (11.6)	smMIPs	46/1209 (3.8)	Amplicon SEQ
**Family 36**								
LR12-345	saliva	3:178938934	c.2333G>A	p.Glu726Lys	42/301 (14)	smMIPs	19/311 (6.1)	Amplicon SEQ
LR12-345f	saliva	3:178938934	c.2333G>A	p.Glu726Lys	0/227 (0)	Amplicon SEQ	–	–
LR12-345m	saliva	3:178938934	c.2333G>A	p.Glu726Lys	0/441 (0)	Amplicon SEQ	–	–
**Family 37**								
LR12-418 (S1)	saliva	3:178938934	c.2333G>A	p.Glu726Lys	38/290 (13.1)	smMIPs	8/238 (3.4)	Amplicon SEQ
LR12-418 (S2)	L tonsil	3:178938934	c.2333G>A	p.Glu726Lys	18/475 (3.8)	smMIPs	0/217 (0)	Amplicon SEQ
LR12-418 (S3)	R tonsil	3:178938934	c.2333G>A	p.Glu726Lys	18/501 (3.6)	smMIPs	0/749 (0)	Amplicon SEQ
**Family 38**								
LR12-431 (S1)	blood	3:178938934	c.2333G>A	p.Glu726Lys	61/1204 (5.4)	smMIPs	28/1206 (2.3)	Amplicon SEQ
LR12-431 (S2)	saliva	3:178938934	c.2333G>A	p.Glu726Lys	4/35 (11.4)	smMIPs	61/1323 (4.6)	Amplicon SEQ
LR12-431 (S3)	buccal swab	3:178938934	c.2333G>A	p.Glu726Lys	5/56 (8.9)	smMIPs	0/128 (0)*	Amplicon SEQ

LR12-431f	blood	3:178938934	c.2333G>A	p.Glu726Lys	0/948 (0)	Amplicon SEQ	–	–
LR12-431m	blood	3:178938934	c.2333G>A	p.Glu726Lys	0/279 (0)	Amplicon SEQ	–	–
**Family 39**								
LR13-051	skin FB	3:178938934	c.2333G>A	p.Glu726Lys	68/995 (6.8)	smMIPs	53/263 (20.2)	Amplicon SEQ
LR13-051f	saliva	3:178938934	c.2333G>A	p.Glu726Lys	0/597 (0)	Amplicon SEQ	–	–
LR13-051m	saliva	3:178938934	c.2333G>A	p.Glu726Lys	0/375 (0)	Amplicon SEQ	–	–
**Family 40**								
LR13-119	saliva	3:178938934	c.2333G>A	p.Glu726Lys	7/95 (7.4)	smMIPs	failed x3	Amplicon SEQ
LR13-119f	saliva	3:178938934	c.2333G>A	p.Glu726Lys	0/56 (0)	Amplicon SEQ	–	–
LR13-119m	saliva	3:178938934	c.2333G>A	p.Glu726Lys	0/624 (0)	Amplicon SEQ	–	–
**Family 41**								
LR15-246	blood	3:178938934	c.2333G>A	p.Glu726Lys	25/42 (59.5)	WES	–	–
LR15-246f	blood	3:178938934	c.2333G>A	p.Glu726Lys	ND (0)	WES	–	–
LR15-246m	blood	3:178938934	c.2333G>A	p.Glu726Lys	ND (0)	WES	–	–
**Kinase domain (AA 797-1068) N = 31**								
**Family 42**								
LR13-045 (S1)	saliva	3:178947865	c.2740G>A	p.Gly914Arg	0/27 (0)	smMIPs	6/1246 (0.5)	Amplicon SEQ
LR13-045 (S2)	skin FB (affected)	3:178947865	c.2740G>A	p.Gly914Arg	88/575 (15.3)	smMIPs	264/2142 (12)	Amplicon SEQ
LR13-045 (S3)	skin FB (unaffected)	3:178947865	c.2740G>A	p.Gly914Arg	55/1033 (5.3)	smMIPs	70/1684 (4)	Amplicon SEQ

LR13-045f	saliva	3:178947865	c.2740G>A	p.Gly914Arg	0/1210 (0)	Amplicon SEQ	–	–
LR13-045m	saliva	3:178947865	c.2740G>A	p.Gly914Arg	0/12 (0)	Amplicon SEQ	–	–
**Family 43**								
LR13-038 (S1)	blood	3:178947865	c.2740G>A	p.Gly914Arg	50/1531 (3.3)	smMIPs	97/3227 (3)	Amplicon SEQ
LR13-038 (S2)	skin FB	3:178947865	c.2740G>A	p.Gly914Arg	305/658 (46.4)	smMIPs	949/2412 (39)	Amplicon SEQ
LR13-038f	blood	3:178947865	c.2740G>A	p.Gly914Arg	0/2613 (0)	Amplicon SEQ	–	–
LR13-038m	blood	3:178947865	c.2740G>A	p.Gly914Arg	0/2122 (0)	Amplicon SEQ	–	–
**Family 44**								
LR13-050 (S1)	blood	3:178947865	c.2740G>A	p.Gly914Arg	68/995 (6.8)	smMIPs	185/2556 (7.2)	Amplicon SEQ
LR13-050 (S2)	saliva	3:178947865	c.2740G>A	p.Gly914Arg	114/633 (18)	smMIPs	265/2073 (12.8)	Amplicon SEQ
LR13-050f (S1)	blood	3:178947865	c.2740G>A	p.Gly914Arg	0/2359 (0)	Amplicon SEQ	–	–
LR13-050f (S2)	saliva	3:178947865	c.2740G>A	p.Gly914Arg	0/2306 (0)	Amplicon SEQ	–	–
LR13-050m (S1)	blood	3:178947865	c.2740G>A	p.Gly914Arg	0/2027 (0)	Amplicon SEQ	–	–
LR13-050m (S2)	saliva	3:178947865	c.2740G>A	p.Gly914Arg	0/2587 (0)	Amplicon SEQ	–	–
**Family 45**								
LR12-130	saliva	3:178947865	c.2740G>A	p.Gly914Arg	28/150 (18.7)	smMIPs	293/1526 (19.2)	Amplicon SEQ
LR12-130f	saliva	3:178947865	c.2740G>A	p.Gly914Arg	0/2083 (0)	Amplicon SEQ	–	–
LR12-130m	saliva	3:178947865	c.2740G>A	p.Gly914Arg	0/2820 (0)	Amplicon SEQ	–	–
**Family 46**								

LR12-327	saliva	3:178947865	c.2740G>A	p.Gly914Arg	28/455 (6.2)	smMIPs	134/2826 (4.7)	Amplicon SEQ
LR12-327m	saliva	3:178947865	c.2740G>A	p.Gly914Arg	0/2552 (0)	Amplicon SEQ	–	–
**Family 47**								
LR12-343 (S1)	blood	3:178947865	c.2740G>A	p.Gly914Arg	743/2494 (30)	Amplicon SEQ	68/137 (49.6)	WES
LR12-343 (S2)	saliva	3:178947865	c.2740G>A	p.Gly914Arg	127/236 (53.8)	smMIPs	1494/3320 (45)	Amplicon SEQ
LR12-343f	saliva	3:178947865	c.2740G>A	p.Gly914Arg	0/312 (0)	WES	0/3329 (0)	Amplicon SEQ
LR12-343m	saliva	3:178947865	c.2740G>A	p.Gly914Arg	0/178 (0)	WES	0/1857 (0)	Amplicon SEQ
**Family 48**								
LR12-383	saliva	3:178947865	c.2740G>A	p.Gly914Arg	37/176 (21)	smMIPs	288/2422 (12)	Amplicon SEQ
LR12-383f	saliva	3:178947865	c.2740G>A	p.Gly914Arg	0/1748 (0)	Amplicon SEQ	–	–
LR12-383m	saliva	3:178947865	c.2740G>A	p.Gly914Arg	0/2694 (0)	Amplicon SEQ	–	–
**Family 49**								
LR13-047	blood	3:178947865	c.2740G>A	p.Gly914Arg	91/865 (10.5)	smMIPs	183/2303 (8)	Amplicon SEQ
**Family 50**								
LR11-382	blood	3:178947865	c.2740G>A	p.Gly914Arg	68/3864 (1.8)	smMIPs	40/2815 (1.4)	Amplicon SEQ
**Family 51**								
LR15-337 (S1)	blood	3:178947865	c.2740G>A	p.Gly914Arg	158/2498 (6.3)	Agilent SS	–	–
LR15-337 (S2)	skin FB	3:178947865	c.2740G>A	p.Gly914Arg	451/1668 (10.3)	Agilent SS	–	–

**Family 52**								
LR13-294	blood	3:178948044	c.2816A>G	p.Asp939Gly	94/291 (32.3)	smMIPs	50/181 (27.6)	Amplicon SEQ
LR13-294f	blood	3:178948044	c.2816A>G	p.Asp939Gly	0/6 (0)	Amplicon SEQ	–	–
LR13-294m	blood	3:178948044	c.2816A>G	p.Asp939Gly	0/26 (0)	Amplicon SEQ	–	–
**Family 53**								
LR12-340	saliva	3:178948100	c.2872C>A	p.Gln958Lys	45/98 (45)	smMIPs	8/31 (25.8)	Amplicon SEQ
LR12-340f	saliva	3:178948100	c.2872C>A	p.Gln958Lys	0/30 (0)	Amplicon SEQ	–	–
LR12-340m	saliva	3:178948100	c.2872C>A	p.Gln958Lys	0/250 (0)	Amplicon SEQ	–	–
**Family 54**								
LR06-337 (S1)	blood	3:178948136	c.2908G>A	p.Glu970Lys	246/1260 (19.5)	smMIPs	0/47 (0)	Amplicon SEQ
LR06-337 (S2)	blood	3:178948136	c.2908G>A	p.Glu970Lys	0/72 (0)	Amplicon SEQ	–	–
LR06-337 (S3)	LB	3:178948136	c.2908G>A	p.Glu970Lys	39/251 (15.5)*	smMIPs	0/18 (0)	Amplicon SEQ
LR06-337f (S1)	LB	3:178948136	c.2908G>A	p.Glu970Lys	0/103 (0)	Amplicon SEQ	–	–
LR06-337f (S2)	blood	3:178948136	c.2908G>A	p.Glu970Lys	0/125 (0)	Amplicon SEQ	–	–
LR06-337m (S1)	LB	3:178948136	c.2908G>A	p.Glu970Lys	0/62 (0)	Amplicon SEQ	–	–
LR06-337m (S2)	blood	3:178948136	c.2908G>A	p.Glu970Lys	0/8 (0)	Amplicon SEQ	–	–
**Family 55**								
LR13-108	saliva	3:178952006	c.3061T>C	p.Tyr1021His	165/409 (40.3)	smMIPs	703/2100 (33.5)	Amplicon SEQ
LR13-108f (S1)	blood	3:178952006	c.3061T>C	p.Tyr1021His	0/2932 (0)	Amplicon SEQ	–	–

LR13-108f (S2)	saliva	3:178952006	c.3061T>C	p.Tyr1021His	0/2754 (0)	Amplicon SEQ	–	–
LR13-108m (S1)	blood	3:178952006	c.3061T>C	p.Tyr1021His	0/1754 (0)	Amplicon SEQ	–	–
LR13-108m (S2)	saliva	3:178952006	c.3061T>C	p.Tyr1021His	0/2798 (0)	Amplicon SEQ	–	–
**Family 56**								
LR11-081	skin FB (affected)	3:178952018	c.3073A>G	p.Thr1025Ala	472/1223 (38.6)	Agilent SS	–	–
**Family 57**								
LR13-169	saliva	3:178952048	c.3103G>A	p.Ala1035Thr	32/137 (23.4)	smMIPs	345/1616 (21.4)	Amplicon SEQ
LR13-169f	saliva	3:178952048	c.3103G>A	p.Ala1035Thr	0/2362 (0)	Amplicon SEQ	–	–
LR13-169m	saliva	3:178952048	c.3103G>A	p.Ala1035Thr	0/4523 (0)	Amplicon SEQ	–	–
**Family 58**								
LR12-462	skin FB (affected)	3:178952048	c.3103G>A	p.Ala1035Thr	223/411 (54.3)	smMIPs	1060/2214 (47.9)	Amplicon SEQ
**Family 59**								
LR12-486	skin FB (affected)	3:178952049	c.3104C>T	p.Ala1035Val	15/55 (27.3)	smMIPs	223/1587 (14.1)	Amplicon SEQ
LR12-486f	blood	3:178952049	c.3104C>T	p.Ala1035Val	0/2100 (0)	Amplicon SEQ	–	–
LR12-486m	blood	3:178952049	c.3104C>T	p.Ala1035Val	0/3737 (0)	Amplicon SEQ	–	–
**Family 60**								

LR12-328	saliva	3:178952074	c.3129G>T	p.Met1043Ile	110/686 (16)	smMIPs	225/1646 (13.8)	Amplicon SEQ
LR12-328f (S1)	saliva	3:178952074	c.3129G>T	p.Met1043Ile	0/2311 (0)	Amplicon SEQ	–	–
LR12-328f (S2)	blood	3:178952074	c.3129G>T	p.Met1043Ile	0/1738 (0)	Amplicon SEQ	–	–
LR12-328m (S1)	saliva	3:178952074	c.3129G>T	p.Met1043Ile	0/66 (0)	Amplicon SEQ	–	–
LR12-328m (S2)	blood	3:178952074	c.3129G>T	p.Met1043Ile	0/834 (0)	Amplicon SEQ	–	–
**Family 61**								
LR12-203 (S1)	blood	3:178952074	c.3129G>T	p.Met1043Ile	205/2850 (7.2)	smMIPs	92/2346 (3.9)	Amplicon SEQ
LR12-203 (S2)	saliva	3:178952074	c.3129G>T	p.Met1043Ile	89/789 (11.3)	smMIPs	105/2371 (4.4)	Amplicon SEQ
LR12-203f (S1)	blood	3:178952074	c.3129G>T	p.Met1043Ile	0/2147 (0)	Amplicon SEQ	–	–
LR12-203f (S2)	saliva	3:178952074	c.3129G>T	p.Met1043Ile	0/1543 (0)	Amplicon SEQ	–	–
LR12-203m (S1)	blood	3:178952074	c.3129G>T	p.Met1043Ile	0/1977 (0)	Amplicon SEQ	–	–
LR12-203m (S2)	saliva	3:178952074	c.3129G>T	p.Met1043Ile	0/2584 (0)	Amplicon SEQ	–	–
**Family 62**								
LR15-043	skin FB (affected)	3:178952074	c.3129G>T	p.Met1043Ile	143/471 (30.4)	Agilent SS	–	–
**Family 63**								
LR06-336	blood	3:178952074	c.3129G>T	p.Met1043Ile	113/3502 (3.2)	smMIPs	38/1663 (2.3)	Amplicon SEQ
**Family 64**								
LR11-039 (S1)	blood	3:178952074	c.3129G>T	p.Met1043Ile	82/2964 (2.8)	smMIPs	73/3862 (1.9)	Amplicon SEQ
LR11-039 (S2)	saliva	3:178952074	c.3129G>T	p.Met1043Ile	69/583 (11.8)	smMIPs	193/2290 (8.4)	Amplicon SEQ

LR11-039f (S1)	blood	3:178952074	c.3129G>T	p.Met1043Ile	0/1908 (0)	Amplicon SEQ	–	–
LR11-039f (S2)	saliva	3:178952074	c.3129G>T	p.Met1043Ile	0/3180 (0)	Amplicon SEQ	–	–
LR11-039m (S1)	blood	3:178952074	c.3129G>T	p.Met1043Ile	0/2530 (0)	Amplicon SEQ	–	–
LR11-039m (S2)	saliva	3:178952074	c.3129G>T	p.Met1043Ile	0/2467 (0)	Amplicon SEQ	–	–
**Family 65**								
LR12-010 (S1)	blood	3:178952074	c.3129G>T	p.Met1043Ile	430/3318 (13)	smMIPs	156/1565 (10)	Amplicon SEQ
LR12-010 (S2)	skin FB	3:178952074	c.3129G>T	p.Met1043Ile	724/2493 (29)	smMIPs	422/2187 (19.3)	Amplicon SEQ
LR12-010f	blood	3:178952074	c.3129G>T	p.Met1043Ile	0/2155 (0)	Amplicon SEQ	–	–
LR12-010m	blood	3:178952074	c.3129G>T	p.Met1043Ile	0/839 (0)	Amplicon SEQ	–	–
**Family 66**								
LR14-300	blood	3:178952084	c.3139C>T	p.His1047Tyr	8/392 (2)	Agilent SS	–	–
LR14-300	skin FB (affected)	3:178952084	c.3139C>T	p.His1047Tyr	673/1131 (59.5)	Agilent SS	–	–
**Family 67**								
LR11-285 (S1)	blood	3:178952084	c.3139C>T	p.His1047Tyr	209/4903 (4.3)	smMIPs	282/1496 (18.9)	Amplicon SEQ
LR11-285 (S2)	saliva	3:178952084	c.3139C>T	p.His1047Tyr	634/2496 (25.4)	smMIPs	84/2880 (2.9)	Amplicon SEQ
LR11-285 (S3)	occipital bone	3:178952084	c.3139C>T	p.His1047Tyr	53/8033 (0.7)	smMIPs	15/2983 (0.5)	Amplicon SEQ
LR11-285f	saliva	3:178952084	c.3139C>T	p.His1047Tyr	0/2556 (0)	Amplicon SEQ	–	–
LR11-285m	saliva	3:178952084	c.3139C>T	p.His1047Tyr	0/1695 (0)	Amplicon SEQ	–	–

**Family 68**								
LR13-172	blood	3:178952085	c.3140A>G	p.His1047Arg	51/4074 (1.3)	smMIPs	30/2182 (1.4)	Amplicon SEQ
**Family 69**								
LR13-265a1 (S1)	skin FB #1	3:178952085	c.3140A>G	p.His1047Arg	58/1362 (4.2)	smMIPs	0/183 (0)	Amplicon SEQ
LR13-265a1 (S2)	skin FB #2	3:178952085	c.3140A>G	p.His1047Arg	0/2854 (0)	smMIPs	0/1682 (0)	Amplicon SEQ
LR13-265a1 (S3)	skin FB #3	3:178952085	c.3140A>G	p.His1047Arg	0/3065 (0)	smMIPs	0/2481 (0)	Amplicon SEQ
LR13-265a1 (S4)	blood	3:178952085	c.3140A>G	p.His1047Arg	1/1341 (0)	smMIPs	114/2689 (4.2)	Amplicon SEQ
LR13-265s1 (S5)	blood	3:178952085	c.3140A>G	p.His1047Arg	0/1672 (0)	Amplicon SEQ	–	–
**Family 70**								
LR06-340	LB	3:178952090	c.3145G>A	p.Gly1049Ser	10/272 (3.7)	smMIPs	19/1819 (1)	Amplicon SEQ
**Family 71**								
LR12-089 (S1)	blood	3:178952152	c.3207A>G	p.stop1069Trp ext*4	424/1904 (22.3)	smMIPs	612/3309 (18.5)	Amplicon SEQ
LR12-089 (S2)	saliva	3:178952152	c.3207A>G	p.stop1069Trp ext*4	106/286 (37.1)	smMIPs	940/2840 (33.1)	Amplicon SEQ

LR12-089f (S1)	blood	3:178952152	c.3207A>G	p.stop1069Trp ext*4	0/2628 (0)	Amplicon SEQ	–	–
LR12-089f (S2)	saliva	3:178952152	c.3207A>G	p.stop1069Trp ext*4	0/1846 (0)	Amplicon SEQ	–	–
LR12-089m (S1)	blood	3:178952152	c.3207A>G	p.stop1069Trp ext*4	0/2399 (0)	Amplicon SEQ	–	–
LR12-089m (S2)	saliva	3:178952152	c.3207A>G	p.stop1069Trp ext*4	0/2015 (0)	Amplicon SEQ	–	–
**Family 72**								
LR11-446	saliva	3:178952152	c.3207A>G	p.stop1069Trp ext*4	60/270 (22.2)	smMIPs	308/1784 (17.3)	Amplicon SEQ
LR11-446f	saliva	3:178952152	c.3207A>G	p.stop1069Trp ext*4	0/1886 (0)	Amplicon SEQ	–	–
LR11-446m	saliva	3:178952152	c.3207A>G	p.stop1069Trp ext*4	0/3107 (0)	Amplicon SEQ	–	–

Abbreviations: WES, whole exome sequencing; SEQ, sequencing; SS, SureSelect.

Supplementary Table 2. Summary of clinical data of *PIK3CA* mutation positive patients by domain (N = 72)

DB#	Sex	Age	Dx	OFC	Weight	Length	SEG OVG	VM Skin	VM Other	CARD	THROM	LYMPH	Digital	ENDO
**PI3K-ABD (AA 16-105) N = 4**														
LR14-323	F	13m	MCAP	+3.5/ +3.5	0/ +2	+2/ +1.5	–	+	+	+	–	–	–	+
LR15-238	F	18m	OVG	+1/ +1	+0.7/ +0.3	0.5/ 0	+ (HH)	+	–	+	+	–	–	–
LR01-060	M	13.5y	MCAP	+6.5/ +7	+2.5/ +0.4	+3/ +1.1	–	+	+	+	–	–	–	–
LR13-359	M	6m	MCAP	+5.5/ +3.5	+0.7/ –2	–0.4/ +0.3	+	–	–	–	–	–	+	–
**Linker region (AA 106-186) N = 5**														
LR11-082	F	3y	OVG	+0.5/ ND	+1.6/ ND	–0.4/ ND	++	++	+	–	–	–	+	–
LR04-078	M	12y4m	MCAP	+5/ +5.5	+2.3/ –1.6	+1.9/ ND	+	+	–	–	–	–	+	–
LR11-397	F	1y1m	MCAP	+3.8/ +2.5	+1.4/ +0.7	+1.3/ –0.5	+ (HH)	+	–	–	–	–	+	–
LR12-001	M	Neonate	MCAP	+2.4/ ND	+0.6/ ND	+1/ ND	–	+	–	–	–	+	+	–
LR12-080	M	4y	MCAP	MEG, ND	ND	ND	+ (HH)	+	–	–	–	–	–	–
**C2 membrane (AA 330-487) N = 18**														
LR15-076	F	5y	MEG	–0.3/	–1.6/	–2/	–	–	–	+	–	–	–	–

				2.5	–2	–1.3								
LR12-365	F	3y3m	MCAP	+5.5/ +4.5	+2/ +0.8	+3.6/ +2.5	+ (HH)	+	+	+	–	–	+	+
LR11-076	F	2y6m	MCAP	+3/ +8.5	+1/+1	+1.3/ –0.7	+ (HH)	+	–	–	–	–	+	–
LR13-036	M	2y	MEG-macrosomia	+6/ +11.9	+6/ +2	+2.5/ –1.6	– RHIZ	–	–	+	–	–	+	+
LR13-264	M	19m	MCAP	+2.5/+3	+1/+2	+2/–1	+ (HH)	+	–	–	–	–	+	–
LR11-374	F	22m	MCAP	ND/+6	ND	ND	+ (HH)	ND	ND	ND	ND	ND	ND	ND
LR11-418	M	9m	MCAP	ND/ +2.5	+2.5/ +3	ND/ +2.5	+ (HH)	+	–	+	–	–	+	–
LR12-131	M	11m	OVG	0/+2	–0.4/0	+2/ +0.3	+ (HH)	+	–	–	–	–	+	–
LR13-328	M	2y	MCAP	+0.5/ MEG	+2/+5	+2/ –1	+ (HH)	+ (KTS)	–	–	+	–	–	–
LR12-382	F	34y	MCAP	ND/+6	+3/ND	+3/ND	+ (HH)	+	–	+	–	+	+	+
LR14-278	F	21m	MCAP	ND	ND	ND	+ (HH)	+	–	+	–	+	+	+
LR11-200	M	2y1m	MCAP	+5/+9	+5/+3	–3/ +2	+ (HH)	+	–	–	–	–	+	–
LR14-358	M	4y	MCAP	+3.8/ +6.5	–0.5/ +1.9	0/ND	–	+	–	–	–	–	–	+
LR11-392	M	14y	OVG	ND/ +0.5	ND/ –0.25	ND/ –1.7	+ (HH)	+	–	–	+	–	–	–

LR12-070	M	4y	OVG-MD	ND/–0.5	ND/+2	ND/–1	+ (HH, L)	+	–	–	–	–	–	–
LR12-184	M	10m	OVG	0/0	ND	0/+0.5	+ (HH)	+	+	+	–	+	+	–
LR12-329	M	5y3m	OVG-MD	0/0	ND	ND	+ (HH)	+	–	–	–	–	–	–
LR13-048	F	20y	MCAP	+4/+9	+0.5/ND	ND	+ (HH)	+	–	–	–	–	–	–
**Helical domain (AA 517-694) N = 4**														
LR12-183	M	2y3m	DMEG-LNSS	+6/+5.5	+1/–1	+2.5/+1.9	–	+ (SN)	–	–	–	–	–	–
LR13-197	F	36d	MEG-DMEG	ND/+1.9	ND	ND	+ (HH)	–	–	–	–	–	–	–
LR12-330	M	23m	MCAP	+4.5/+5.5	ND	ND	+	+	–	–	–	–	+	–
LR12-019	M	12m	MCAP	+1.9/+2	ND	ND	+ (HH, ILM)	+	+	–	–	–	+	–
**Linker region (AA 695-796) N = 10**														
LR09-142	F	13y	MCAP	MEG	>2/–1.75	ND/–2	–	+	–	–	–	–	–	+
LR11-072	F	16m	MCAP	ND/+4	+2/+5.5	+2/+5	+ (HH)	+	–	–	–	–	+	–
LR12-037	M	5y1m	MCAP	+5.8/+7	+3/+0.5	ND/–2	– RHIZ	+	+	–	–	–	–	–
LR12-109	M	10y	MCAP	+2.5/+5	ND	+1.9/–3	+ (HH)	+	–	–	–	–	–	–

LR12-345	M	3y6m	MCAP	+1.5/+3	−0.5/ND	0/ND	+ (HH)	+	−	−	−	−	+	+
LR12-418	F	2y	MCAP	+2/+2.5	+0.7/+1.9	0/−1	+ (HH)	+	−	+	−	−	+	−
LR12-431	F	6y1m	MCAP	ND/+6	+1.9/ND	+2/+2	+ (HH)	+	−	+	−	−	+	−
LR13-051	F	5m (deceased)	MCAP	+4/ND	+2.5/ND	+4/ND	−	+	−	+	−	−	+	−
LR13-119	F	2y5m	MCAP	+2/+2.5	+1.9/0	0/+1.9	+ (HH)	+	+	−	+	−	−	−
LR15-246	M	3y1m	MCAP	+6/+5	+2/+2	+1.5/+2	−	+	−	−	+	−	−	−
**Kinase domain (AA 797-1068) N = 31**														
LR13-045	M	23m	OVG	+2/+1.9	+0.5/ND	+0.5/ND	+ (HH)	+	−	−	−	−	−	−
LR13-038	M	14m	MCAP	+2.5/+7	+0.5/+3.5	+1.9/+1.9	+ (HH)	+	−	−	−	−	−	−
LR13-050	M	15y	MCAP	+4.6/+8	ND	ND	+ (HH)	+	−	−	−	−	+	+
LR12-130	M	4m	MCAP	+4.5/+3.2	ND	ND	+ (LLD)	+	−	−	−	−	+	−
LR12-327	F	15y	MCAP	ND (MEG)	ND/+0.8	ND/−2	+ (LLD)	+	−	−	−	−	+	−

LR12-343	M	11m	MCAP	+5.5/ +6.4	+3.6/ –1.2	+2.9/– 2	+ (LLD)	+	–	+	–	–	+	+
LR12-383	F	34y	MCAP	MEG/ +4.8	+1/+3	ND/ –0.5	+ (HH)	+	–	–	–	+	+	–
LR13-047	F	15y	MCAP	+4.7/ +7.6	ND/ +2.8	ND/ +0.8	+ (HH)	+	–	–	–	–	–	–
LR11-382	F	18y	MCAP	ND/ +4.2	+1.3/ +1.6	ND/ –7.1	+ (HH)	+	–	–	–	–	–	–
LR15-337	M	18m	MCAP	ND (MEG) /+2.5	LGA/ ND	ND	+ (HH) RHIZ	+	+	+	–	–	–	–
LR13-294	M	2y	MCAP	+5.5/ +8	+3.2/ ND	+2.9/ –1.5	ND	+	–	–	–	–	–	–
LR12-340	F	8y	OVG	ND / +1.6	ND / –1	ND / +4.5	–	–	–	–	–	–	+	–
LR06-337	M	33m	MCAP	ND/ +4.9	+1/ +0.7	+1.25/ +0.9	+ (HH)	+	–	+	–	–	+	–
LR13-108	M	7m	MCAP	MEG/ +3	–0.7/0	ND/ –1.5	+	+	–	–	–	–	–	–
LR11-081	F	5y	MCAP	MEG/ +3.3	ND/ 0	ND/ –1.75	ND / +3.3	+	–	–	–	+	–	–
LR13-169	M	2.5y	MCAP	+4.5/ +6.4	+3/ +4	+3.5/ +1.3	+ (HH)	+	–	+	–	–	+	–
LR12-462	M	ND	MCAP	ND	ND	ND	ND	ND	ND	ND	ND	ND	ND	ND

LR12-486	F	11.5 m	OVG	ND/ +0.5	+1/ +1.5	+1.5/ +1.4	+ (HH)	+	–	–	–	–	+	–
LR12-328	F	8m	MCAP	+3.1/ +4.8	–0.7/ ND	ND	+	+	–	–	–	–	–	–
LR12-203	M	10y	MCAP	+2/ +3	+1.75/ ND	+1/ ND	+	+	–	+	–	–	–	–
LR15-043	F	13m	MCAP	+1/+4. 5	+3/ +0.7	+4/ +0	–	+	–	–	–	–	–	+
LR06-336	M	3y	MCAP	–0.5/ +2.3	–1/ +1.1	–0.7 +0.4	+ (LLD)	+	–	–	–	–	+	–
LR11-039	M	23m	MCAP	+5.5/ +4.6	+2/– 0.3	+2/–1	+ (HH)	+	–	–	–	–	–	–
LR12-010	F	2y2m	MCAP	ND/ +4.2	+2/ ND	+2.5/ –0.7	+ (HH)	+	–	–	–	–	–	–
LR14-300	M	19y	MCAP	MEG/ +8.2	ND/ +0.5	ND/ –0.42	+ (LLD, facial)	+	–	–	–	+	–	–
LR13-172	F	10y	MD	ND	ND	ND	+	ND	ND	ND	ND	ND	+	ND
LR13-265	M	5y10 m	OVG-CLOVES	ND	ND/ –0.5	ND/ –2	+	+	ND	ND	ND	ND	+	–
LR06-340	F	10y	MCAP	ND	+0.5/ ND	ND	ND	+	–	ND	ND	ND	–	ND
LR12-089	M	2y	MCAP	MEG/ +3.2	+1.6/ +2.6	ND/ –5	+ (HH)	+	–	–	–	–	+	–
LR11-446	M	5y	MCAP	+4.4/ +4.3	+2.1/ +2.3	+2.6/ +1.3	+ (HH)	+	–	–	–	–	–	–

Abbreviations: CARD, cardiac defects; CLOVES, congenital lipomatous truncal overgrowth, vascular malformations, epidermal nevi, skeletal/spinal anomalies; d, day; Digital, digital anomalies; Dx, diagnosis; ENDO, endocrine problems; F, female; HH, hemihypertrophy; LLD, leg length discrepancy; LYMPH, lymphatic abnormalities; ND, no data; m, month; M, male; MCAP, megalencephaly-capillary malformation; MEG, megalencephaly; OFC, occipito-frontal circumference; OVG, overgrowth; RHIZ, rhizomelic shortening of the extremities; SEG, segmental; THROM, thrombosis; VM, vascular malformations; y, year.

Supplementary Table 3. Detailed clinical data of *PIK3CA* mutation positive patients by domain (N = 72)

DB#	Clinical diagnosis	Overgrowth	Vascular malformations of the skin	Other vascular malformations	Cardiac abnormalities	Thrombosis	Lymphatic malformations	Digital anomalies	Endocrine issues	Other pertinent features
**PI3K-ABD (AA 16-105) N = 4**										
LR14-323[A]	MCAP	–	Facial CapM, hemangiomas (x4; involuted)	Liver hemangioma	Persistent fetal atrial flutter, hydrops, PDA, ASD, MR, TR[A]	–	–	–	Congenital hypothyroidism (maternal Amiodarone treatment)	Frequent respiratory infections (2 pneumonias)
LR15-238	OVG	Hemihypertrophy (mild)	Facial CapM, diffuse CapM	–	Perinatal bradycardia, transient DCM	Non-occlusive venous thrombus in L IJV	–	–	–	–
LR01-060	MCAP	–	Facial CapM, diffuse CapM	Hemangioma a beneath cheek (regressed), chronic subdural hematoma	ASD, VSD, vascular ring, transient tachyarrhythmias	–	–	–	–	Significant apnea, ventilator dependent until 4y

LR13-359	MCAP	Hemihypertrophy	Facial CapM, prominent venous network	–	–	–	–	Syn (2-3 toe, bilateral)	–	–
**Linker region (AA 106-186) N = 5**										
LR11-082	OVG	Diffuse asymmetric OVG, MD	CapM (extensive, diffuse)	Subcutaneous hemangioma	–	–	–	Poly (R foot), syn (2-3 R hand, 2-3-4 toe bilat)	–	Severe connective tissue laxity, multiple joint dislocations at birth
LR04-078	MCAP	Overgrowth of R arm and hand	Facial CapM, CapM (multiple extensive)	–	–	–	–	Poly (R hand, L foot), syn (2-3-4 R hand, 2-3 bilateral foot, 4-5 L foot)	–	–
LR11-397	MCAP	Hemihypertrophy (L leg)	Facial CapM, hyperpigmentation	–	–	–	–	Poly (R hand), syn (2-3-4 L hand, 2-3 R	–	–

			along linea alba					foot, 2-3-4 L foot)		
LR12-001	MCAP	–	CapM (diffuse)	–	–	–	In utero chylothorax, (s/p in utero thoracocentesis at 23+3w of gestation)	Poly (feet bilat)	–	–
LR12-080	MCAP	Hemihypertrophy (L, face and body)	CapM (diffuse, regressed with age)	–	–	–	–	–	–	–
**C2 membrane (AA 330-487) N = 18**										
LR15-076	MEG	–	–	–	Small PFO	–	–	–	–	Pectus excavatum
LR12-365	MCAP	Hemihypertrophy (L, face and body)	Facial CapM, diffuse CapM	Hemangioma (lower spine)	Aorto-pulmonary collaterals, persistent L SVC, MAT	–	–	Poly (L hand), syn (2-3 L foot)	GH deficiency, hypothyroidism	–
LR11-076	MCAP	Hemihypertrophy (L, face and body)	Facial CapM, diffuse CapM	–	–	–	–	Syn (2-3 bilat toe)	–	–

LR13-036[B]	MEG-macrosomia	–	–	–	L aortic arch, PFO, VSD, R atrial dilatation, pulmonary HTN	–	–	Poly (R hand)	Hypoglycemia (recurrent)	Recurrent infections, omphalocele, organomegaly, laryngeal stenosis, rhizomelic shortening of the extremities
LR13-364	MCAP	Hemihypertrophy	Facial CapM, diffuse CapM	–	–	–	–	Syn (2-3 bilat toe), sandal-gap toes	–	–
LR11-374	MCAP	Hemihypertrophy	ND	ND	ND	ND	ND	ND	ND	ND
LR11-418	MCAP	Hemihypertrophy	Facial CapM, diffuse CapM	–	ASD, VSD	–	–	Syn (2-3 bilat toe)	–	–
LR12-131	OVG	Hemihypertrophy	Facial CapM, diffuse CapM	–	–	–	–	Syn (2-3 bilat toe)	–	–

LR13-328	MCAP	Mild lipoatrophy of L arm, macroglossia	Facial CapM, diffuse CapM	–	–	–	–	–	Unexplained episodes of lethargy, hypoglycemia workup negative	–
LR12-382	MCAP	Hemihypertrophy (R face, body)	CapM, prominent veins	–	–	Hypercoagulable state, DVTs, deep cerebral venous thrombosis, CVA	–	Syn (2-3 bilat toe)	–	–
LR14-278	MCAP	Hemihypertrophy (L face, body)	Facial CapM	–	Vascular ring, R aortic arch, aberrant L SCA, paramembranous VSD, s/p surgical correction	–	Chylothorax	Poly (L hand, R foot)	Mild hypoglycemia at birth (resolved)	–
LR11-200	MCAP	Hemihypertrophy	Facial CapM, CapM,	–	–	–	–	Syn (2-3 bilat toe)	–	–

			linear hyperpigmentation							
LR14-358[C]	MCAP	–	Small hemangioma on nose (resolved)	–	–	–	–	–	Ketotic hypoglycemia	–
LR11-392	MCAP	Hemihypertrophy (4.5 cm discrepancy)	CapM (face and legs)	Narrow L renal artery, narrow L internal carotid artery	–	CVA at 4.5 years (involving basal nuclei), recurrent TIA from 4.5y	–	–	–	Bilateral glaucoma from 2y
LR12-070	OVG-MD	Hemihypertrophy, lipomatous overgrowth on trunk, MD (resected)	CapM (diffuse) with underlying lipomas, EN	–	–	–	–	–	–	–
LR12-184	OVG	Hemihypertrophy (L face, body),	Diffuse CapM, prominent	Liver hemangioma, possible	Persistent PDA	–	Sporadic lymphadenopathy,	Syn (2-3 R toe)	–	–

		possible lipomatous overgrowth	veins, fading hemangioma on upper lip	throat hemangioma			suggestive of lymphatic dysregulation			
LR12-329	OVG-MD	Hemihypertrophy, MD, 3 cutaneous lipomas	CapM (diffuse, reticulated)	–	–	–	–	–	–	–
LR13-048	MCAP	Hemihypertrophy	Facial CapM, diffuse CapM	–	–	–	–	–	–	–
**Helical domain (AA 517-694) N = 4**										
LR12-183	DMEG-LNSS	–	Multiple sebaceous nevi	–	–	–	–	–	–	Complex cortical malformations, cerebellar hypoplasia
LR13-197	MEG-DMEG	Hemihypertrophy (R face and extremities)	–	–	–	–	–	–	–	Dysplastic megalencephaly

LR12-330	MCAP	Facial asymmetry	CapM (multiple, diffuse)	–	–	–	–	Poly (hands), syn (2-3 R, 2-3-4 L foot)	–	–
LR12-019	MCAP	Hemihypertrophy, asymmetric abdominal wall thickness (infiltrating lipomatosis)	Facial CapM, diffuse CapM	Dilated R optic central vein	–	–	–	Syn (2-3 R foot, 2-3-4 L foot)	–	Bilateral enophthalmos, bilateral hearing loss
**Linker region (AA 695-796) N = 10**										
LR09-142	MCAP	–	Facial CapM, diffuse CapM	–	–	–	–	Syn (2-3 toes)	Transient hypoglycemia at birth	–
LR11-072	MCAP	Hemihypertrophy (R, mild)	CapM (face)	–	–	–	–	Syn (2-3-4 R hand)	–	–
LR12-037	MCAP	–	Facial CapM, diffuse CapM	–	–	–	–	–	–	Rhizomelic shortening of the extremities

LR12-109	MCAP	Hemihypertrophy	Facial CapM, diffuse CapM	–	–	–	–	–	–	–
LR12-345	MCAP	Hemihypertrophy (mild)	Facial CapM, diffuse CapM	–	–	–	–	Syn (2-3-4 bilat toe)	Neonatal hypoglycemia, primary hypothyroidism	–
LR12-418	MCAP	Hemihypertrophy, cerebral asymmetry without cortical dysplasia	Facial CapM, diffuse CapM	–	PFO, atrial septal aneurysm	–	–	Macrodactyly (2nd R, 2-3 L)	–	–
LR12-431	MCAP	Hemihypertrophy (L)	Facial CapM, diffuse CapM	–	ASD	–	–	Syn (2-3 R foot)	–	–
LR13-051	MCAP	–	Facial CapM, diffuse CapM	–	Tetralogy of Fallot, pulmonary atresia, inoperable,	–	–	Syn (2-3-4 L hand, 2-3-4 bilat feet)	–	–

					deceased from cardiac failure					
LR13-119	MCAP	Hemihypertrophy (L body)	Facial CapM, CapM (x1)	Cavernous hemangioma on left chest, with focal thrombosis	–	–	–	–	–	–
LR15-246	MCAP	–	Facial CapM	–	VSD (perimembranous causing stenosis of the RU, RL, LU PVs) s/p cardiac surgery	–	–	–	–	Autism spectrum disorder
**Kinase domain (AA 797-1068) N = 31**										
LR13-045	OVG	Hemihypertrophy	CapM (diffuse)	–	–	–	–	–	–	–
LR13-038	MCAP	Hemihypertrophy (L)	Facial CapM, diffuse CapM, nevi	–	–		–	–	–	–

LR13-050	MCAP	Hemihypertrophy (mild)	Facial CapM, diffuse CapM	–	–	–	–	–	Hypothalamic hypothyroidism	–
LR12-130	MCAP	Hemihypertrophy (lower limb)	Facial CapM (multiple)	–	Dilated LV	–	–	Poly (feet bilat)	–	SNHL (unilateral), laryngomalacia
LR12-327	MCAP	Leg length discrepancy (s/p surgery)	Facial CapM, diffuse CapM (resolved)	–	–	–	–	Syn (2-3 toe bilat)	–	–
LR12-343	MCAP	Leg length discrepancy	Facial CapM, diffuse CapM	–	Large PDA, PFO, dilated coronary sinus with large L SVC	–	–	Poly (R hand)	GH deficiency, on GH injections	–
LR12-383	MCAP	Hemihypertrophy	Multiple nevi, diffuse CapM	–	–	–	Lymphatic insufficiency with lymphedema of the legs, wears stockings	Syn (2-3 L foot)	–	–

							bilaterally and compression bump on legs BI			
LR13-047	MCAP	Hemihypertrophy	Diffuse CapM	–	–	–	–	–	–	–
LR11-382[D]	MCAP	Hemihypertrophy	Facial CapM, diffuse CapM	–	–	–	–	–		–
LR15-337	MCAP	Hemihypertrophy, thick gums	Facial CapM, diffuse CapM	Bilateral optic disc vascular tortuosity	Small muscular VSD and PDA at birth	–	–	Syn (2-3 toe bilat)	–	Rhizomelic shortening of the extremities
LR13-294	MCAP	ND	Facial CapM	–	–	–	–	–	–	Splenic cysts (2), connective tissue laxity
LR12-340	OVG	–	–	–	–	–	–	Syn (2-3 toe bilat)	–	–
LR06-337	MCAP	Hemihypertrophy	Facial CapM	–	Atrial flutter and tachycardia	–	–	Syn (2-3 toe bilat)	–	–

LR13-108	MCAP	Hands, bilateral lower limbs, left hemiface, left upper limb, spleen clinically palpable	Facial CapM, diffuse CapM (multiple, large)	–	–	–	–	–	–	–
LR11-081	MCAP	Right leg larger than L, overall lower body larger than upper	CapM (multiple, large)	–	–	–	History of neonatal pleural and pericardial effusions (resolved)	–	–	Connective tissue laxity
LR13-169	MCAP	Hemihypertrophy	Diffuse CapM	–	VSD	–	–	Syn (2-3 toe bilat)	–	Neonatal hypocalcemia, hyponatremia, hypokalemia (resolved)
LR12-462	MCAP	ND	ND	ND	ND	ND	ND	ND	ND	ND
LR12-486	OVG	Hemihypertrophy	Hemangiomas	–	–		–	Syn (2-3 toe bilat)	–	–

			(forehead, lip, nuchal region, midline thoracic and sacral regions), diffuse CapM							
LR12-328	MCAP	Face, LLE	CapM (forehead, philtrum, R parieto-occipital area, perianal, sacral areas), reticulated CapM limbs	–	–	–	–	–	–	–
LR12-203	MCAP	Tongue	Facial CapM, diffuse CapM, linear	–	Atrial dilatation, atrial flutter in the	–	–	–	–	Marked joint hypermobility (8/9 Beighton

			hyperpigmented streaks of arms/legs following lines of Blaschko		neonatal period					criteria), hyperelastic skin, bilateral hip dislocation, status post knee surgery for subluxation
LR15-043	MCAP	Hemihypertrophy	Facial CapM, diffuse CapM	–	–	–	–	–	Transient neonatal hypoglycemia	–
LR06-336	MCAP	Leg length discrepancy	CapM (diffuse), Hemangiomas (buttocks, trunk, back)	–	–	–	–	Syn (2-3 toe bilat)	–	–
LR11-039	MCAP	Hemihypertrophy	CapM (diffuse)	–	–	–	–	–	–	–

LR12-010	MCAP	Hemihypertrophy	Facial CapM (multiple), subtle reticular CapM over legs, few hemangiomas (R arm, thorax, abdomen), CuM (lower extremities)	–	–	–	–	–	–	–
LR14-300	MCAP	Facial asymmetry, leg length discrepancy	Multiple CapM (diffuse), small hemangiomas over scalp	–	–	–	Recurrent pleural effusions and edema of unknown etiology	–	–	–
LR13-172	MD	MD (L>R) s/p surgical excision	ND	ND	ND	–	ND	MD (L>R) s/p surgical excision		ND
LR13-265	OVG-CLOVES	Face and trunk asymmetry	Prominent venous network in	–	–	–	–	MD, sandal gap toes	–	Cryptorchidism

		with massive lipomatous tissue, verrucous overgrowth on the tongue, MD	the abdominal area							
LR06-340	MCAP	ND	ND	ND	ND	–	ND	Syn (2-3 toe bilat)	–	ND
LR12-089	MCAP	Hemihypertrophy	Facial CapM, diffuse CapM	–	–	–	–	Syn (2-3 toe bilat)	–	–
LR11-446	MCAP	Hemihypertrophy	Facial CapM, diffuse CapM	–	–	–	–	–	–	–

Abbreviations:
ASD, atrial septal defect; CapM, capillary malformation; d, day; DCM, dilated cardiomyopathy; DVT, deep vein thrombosis; EN, epidermal nevi; IJV, internal jugular vein; LU, left upper; m, month; MAT, multi-focal trial tachycardia; MCAP, megalencephaly-capillary malformation syndrome; MD, macrodactyly; MR, mitral valve regurgitation; ND, no data; OVG, overgrowth; PDA, patent ductus arteriosus; PFO, patent foramen ovale; poly, polydactyly; PV, pulmonary veins; RL, right lower; RU, right upper; SNHL, sensorineural hearing loss; syn, syndactyly; TR, tricuspid valve regurgitation; y, year; SVC, superior vena cava; VSD; ventricular septal defect.

**Other pertinent clinical data:**
[A]**LR14-323:** This patient had persistent fetal atrial flutter with a 2:1 block and poor ventricular function. Mother was treated with sotalol, flecainide, amiodarone, and digoxin. Fetal echocardiogram showed dilated right ventricle, mild mitral regurgitation, mild-moderate Tricuspid valve regurgitation, unrestrictive patent ductus arteriosus, and atrial septal defect. Fetal tachycardia noted at 29 weeks of gestation and converted to sinus rhythm by esophageal pacing. Repeat Echocardiogram at 36 weeks showed resolution of hydrops but persistent atrial flutter with 2:1 block. Echocardiogram at postnatal day 7 showed dilated right ventricle, mild mitral regurgitation, mild-moderate tricuspid regurgitation, unrestrictive patent ductus arteriosus, atrial septal defect with left to right shunt.
[B]**LR13-036:** This patient had several types of recurrent infections including sinusitis (x20), tracheitis, (x20), pneumonia (x10), multiple episodes of otitis media, and multiple episodes of conjunctivitis.
[C]**LR14-358:** This patient had recurrent hypoglycemia with episodic lethargy. Following a 17 hour fast, his blood glucose level was 2.3 mmol/L. Insulin levels were low at 2 mIU/L, with a normal cortisol level (754 nmol/L), and a low growth hormone response (0.6 ng/mL) without evidence of growth hormone deficiency, but believed to be due to delayed growth hormone response. His motor examination was normal. He had features of Autism Spectrum Disorder.
[D]**LR11-382:** This patient had early onset varicosities at age 20 year and developed thrombosis and cerebrovascular accident of the right brainstem soon after being prescribed oral contraceptives. Her course was complicated by pulmonary embolism as well.
**Note:** Further clinical details are available on previously published patients: LR01-060[52] (patient 3), LR12-001[53], LR12-382 (patient 7)[54], LR13-048 and LR13-050[55] (patients 1 and 2, respectively), LR09-142[56], LR06-337[21] (patient 14), LR13-265[57], LR11-285[34].

Supplementary Table 4. *PIK3CA* primer sequences used for amplicon sequencing

Mutation(s)	Forward Primer	Reverse Primer
p.Trp11Gly	TCAAGTCAAACTATGGAAAATGAGTT	CCTCACGGAGGCATTCTAAA
p.Pro104Leu; P.Gly106Val; p.Arg115Pro	GACGACTTTGTGACCTTCGG	AGAAAGGGACAACAGTTAAGCT
p.Asn345Thr; p.Asp350Asn	TCTTTGTGCAACCTACGTGA	CTAAGGTAGATGGAAGCTCAAGT
p.Cys378Tyr	GGAGAACCAAGCTATATCTGAACAA	GCCCAGGCTGGTCTAAAAA
p.Pro449Thr; p.Glu453Lys; p.Glu453X	CCTTTTGGGGAAGAAAAGTG	GGATTTGATCCAGTAACACCAA
p.Asn515Ser; p.Glu542Lys; p.Glu545Asp	TTGGTTCTTTCCTGTCTCTGAA	TCCATTTTAGCACTTACCTGTGA
p.Ser571Phe	TGGAGAAGTTAGACATGTCAACC	ACCTGGGCTACTTCATCTCT
p.Glu726Lys	CCTATTGTCGTGCATGTGGG	TCTAAACAACTCTGCCCCACT
p.Trp783Leu	GTGTCGAATTATGTCCTCTGCA	ACTCTTCCTTACCATCCCCA
p.Val845Gly	AAGGCAGTAAAGGTCATGCA	ACCTTTCAAGCCGCCTTTG
p.Gly914Arg	ATGGAAACTTGCACCCTGTT	TCCATCGTCTTTCACCATGA
p.Asp939Gly; p.Gln958Lys; p.Glu970Lys	TGGTGAAAGACGATGGACAAG	GCTCACCTCTCAAATTCTCTTGT
p.Tyr1021His; p.Ala1035Thr; p.Met1043Ile; p.His1047Tyr; p.Gly1049Ser; p.stop1069Trpext*4	GATGCTTGGCTCTGGAATGC	ACAGTGCAGTGTGGAATCCA
